# Follow the Molecule from Crystal Arthropathy to Comorbidities: The 2024 G-CAN Gold Medal Award Awardee Lecture

**DOI:** 10.3390/gucdd3030017

**Published:** 2025-09-02

**Authors:** Robert Terkeltaub

**Affiliations:** Department of Medicine, Division of Rheumatology, Autoimmunity, and Inflammation, University of California San Diego, La Jolla, CA 92093, USA

**Keywords:** gout hyperuricemia, calcium pyrophosphate, CPPD monosodium urate crystal, arterial calcification, atherosclerosis, osteoarthritis, chondrocalcinosis, cytokines

## Abstract

Gout and calcium pyrophosphate crystal deposition disease (CPPD) are frequently associated with comorbid disorders, including coronary artery disease and osteoarthritis, in which ectopic calcification with basic calcium phosphate crystals commonly affects arteries and articular cartilage, respectively. Accepting the 2024 G-CAN Gold Medal, I review my research philosophy for translational etiopathogenesis investigation in gout and CPPD, atherosclerosis, responses to arterial injury, and osteoarthritis. Since molecular homeostasis points to pathophysiology and vice versa, I have followed selected molecular players and pathways to phenotypes. Typically, behind each disease target is another target. Illuminating passageways between etiopathogenic pathways is especially productive when using approaches beyond conventional “omics” to reveal the impact of specific post-translational protein modifications, and changes in protein conformation, complex assembly, and interactomes. Highlighting these concepts, I review my past studies on specific molecular pathways, and current perspectives for the following: (i) PP_i_, NPP1, ANKH, and transglutaminase 2 (TG2); (ii) relationships between NPP1, ANKH, Vanin-1 Pantetheinase, and ectopic chondrogenesis; (iii) intersections between adenosine, AMPK, CXCL8 and its receptor CXCR2, the receptor for advanced glycation endproducts (RAGE) and chondrocyte hypertrophy; (iv) lubricin homeostasis and proteolysis; (v) receptor for advanced glycation endproducts (RAGE) and TG2-catalyzed post-translational calgranulin modification; (vi) complement activation and C5b-9 assembly, and the nucleotide-bound conformation of TG2. The inescapable conclusion is that these molecular pathways tightly knit crystal arthropathy with both arterial and osteoarthritis comorbidity.

## 1. Introduction

I am honored to accept the 2025 G-CAN Gold Medal. Invited to write an opinion piece that capsulates my invited awardee lecture, I summarize my research philosophy and accomplishments related to crystal arthropathy and associated comorbidities. In doing so, I add selected historical and current perspectives, including where recent works by others have expanded on the original studies to reshape the field.

## 2. How the Work Began

My work began in 1981; after training at McGill University in Montreal, Canada as a rheumatologist, with fellowship clinical research mentored by John Esdaile, I moved to La Jolla, California to dive into basic lab research. There, my primary mentor was Mark Ginsberg, a rheumatologist whose interest in gout was kindled by fellowship training under Dan McCarty in Chicago. MSU crystals had previously been established to activate complement [1] and the contact system of coagulation [2], and to induce a chemotactic factor from neutrophils [3]. Topical investigation on gout was then progressing to deeper elucidation of the breadth of circulating and cell proteins that bind the monosodium urate (MSU) crystal surface, and how the crystals activate inflammatory cells [4].

My initial studies in crystal disease bumped up against the limitations of the “pre-omics” era of crystal arthropathy research, when there was a conspicuous absence of contemporary research tools to molecularly define and study signal transduction, gene expression, DNA variants, the epigenome, recombinant proteins, and cytokines. Nevertheless, my philosophy from the outset has been to use all the tools at my disposal to follow specific molecules from the bench to preclinical and clinical biology.

## 3. Molecular Targets and Pathways in My Research Philosophy

**Figure 1.**
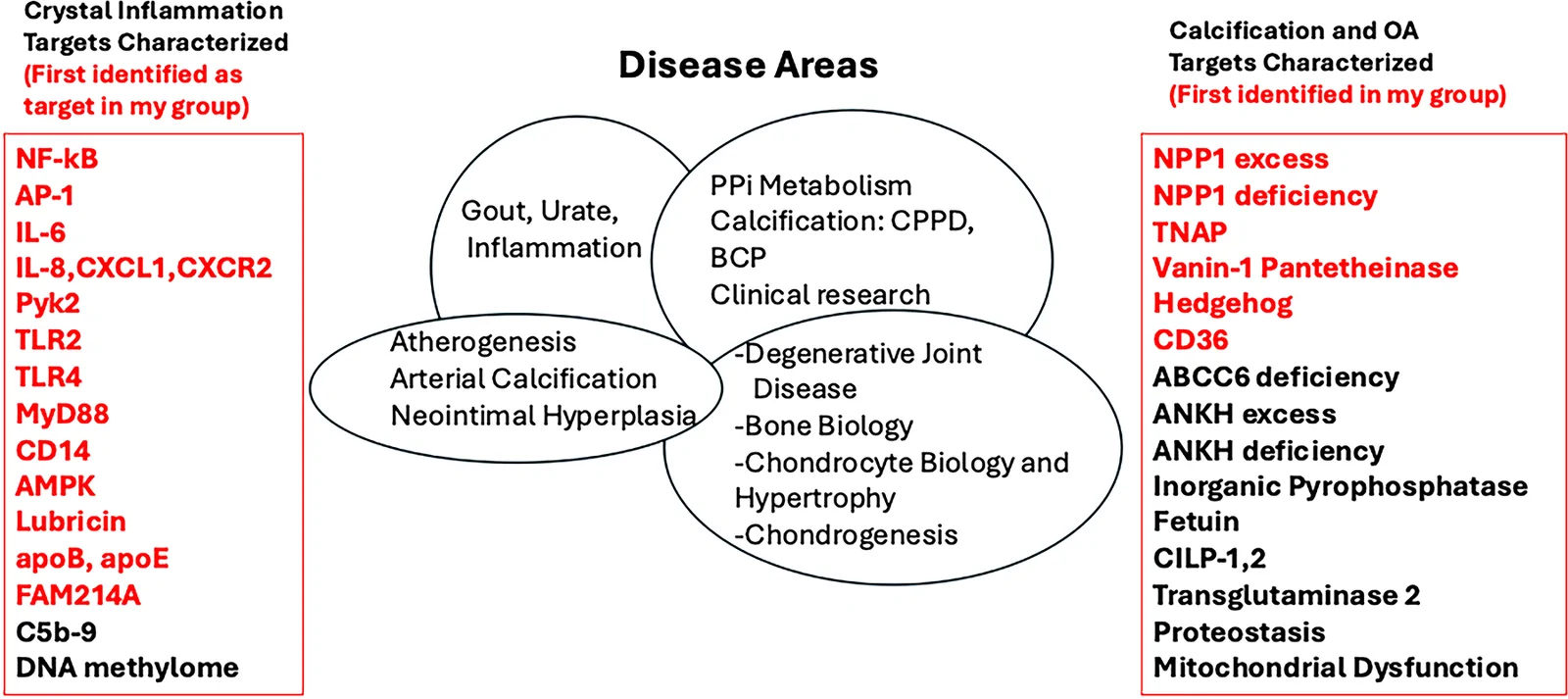
Disease target molecules in crystal arthropathy and pathologic calcification studied in my lab, and in many cases followed to comorbidities. The figure lists pathophysiology targets I have worked on, including those (listed in red, on the left) first identified to be involved in crystal-induced inflammation in my various lab groups and with my collaborators. Similarly listed on the right of the Figure are targets for calcification and osteoarthritis that I seminally identified and/or worked on.

Discovering target molecules and pathways involved in both disease and tissue homeostasis has been the primary focus of my basic-translational research (Figure 1). This work channeled the tenets of my research philosophy and efforts. Furthermore, my group, and global network of collaborators, went beyond gout and other forms of crystal arthropathy to the analysis of specific comorbidities of gout, CPPD, and pathologic calcification associated with deposition of basic calcium phosphate crystals. These studies ranged from degenerative joint disease associated with dysregulated chondrocyte biology to atherogenesis, arterial calcification, and how arteries react to injury in the process of neointimal hyperplasia.

The divine ingenuity of nature created large and small molecules, with NPP1 and PP_i_, respectively, as fine examples [5] for tissue homeostasis, growth, and repair functions that prevent or limit disease. Nature is curiously economical, in that many molecules essential to physiology function in multiple organs, and not only deficiency but also excess of such molecules cause disease in multiple organs; I have been reminded of this time and time again in my own work.

**Figure 2.**
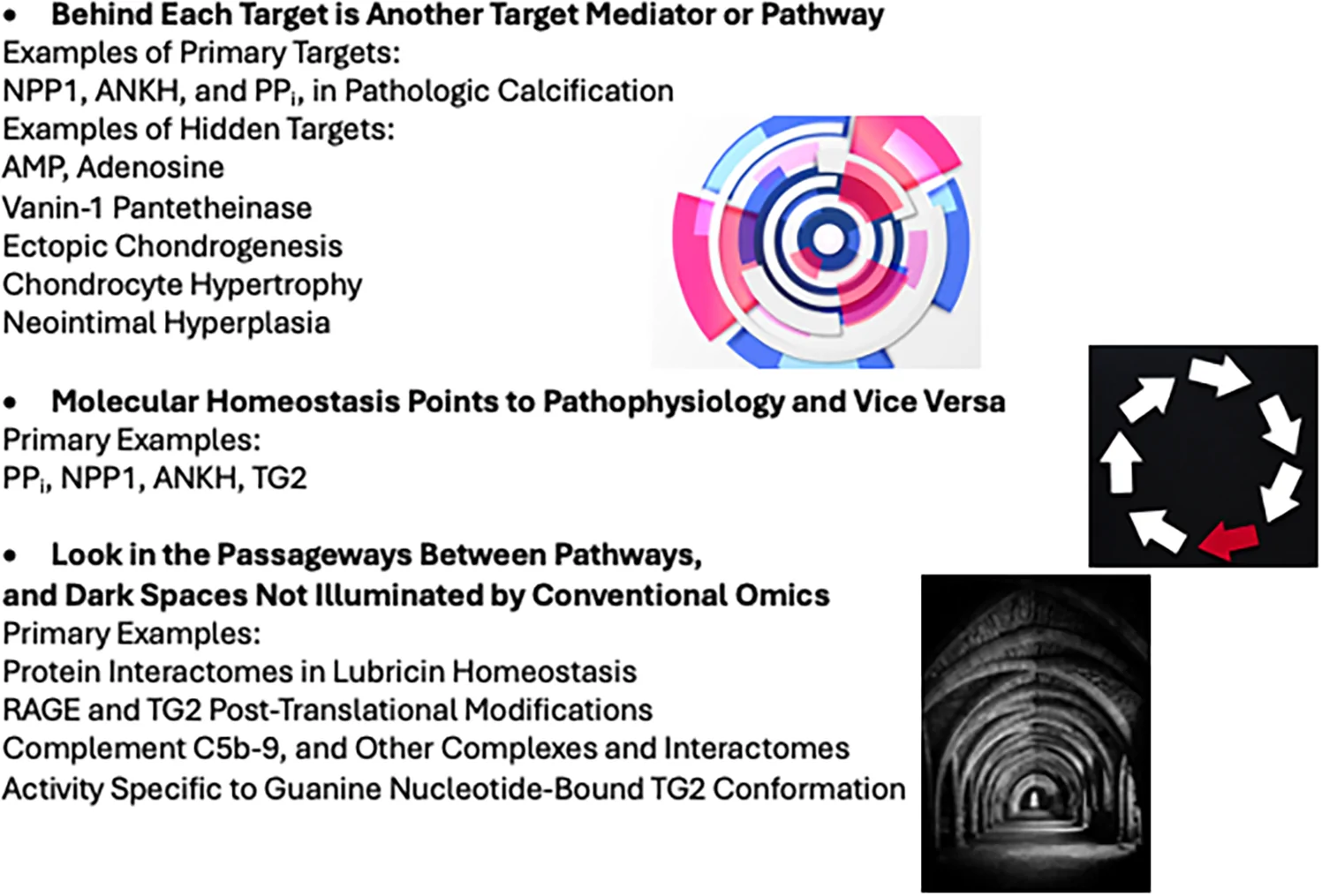
My research philosophy. The Figure schematizes my path in following molecules through to the clinical phenotypes of crystal arthropathies and their comorbidities (including atherosclerosis, arterial and other forms of ectopic calcification, and osteoarthritis). Images in the Figure are licensed free from Freepik and accredited to the image author: Designed by Freepik.

My research philosophy (Figure 2) has been honed by experience in the processes of discoveries highlighted in this review. Importantly, we continually found that behind the primary target we studied was one or more other major target pathways. Combing the passageways between pathways, and the dark spaces not illuminated by conventional omics, allowed us to study protein complexes and interactomes, and highlight biologic effects of post-translational modifications (Figure 2).

## 4. The Approaches Started with Proteomics

My studies began in 1981 with proteomics, before that name was bestowed. I used spots on O’Farrell 2-dimensional gels to identify the MSU crystal-bound proteome of plasma and serum exposure [6]. The proteome bound to MSUcrystals exposed to plasma or serum turned out to be distinct from that of silica [6], and of CPP and basic calcium phosphate crystals [7]. For example, we found that MSU crystals bound C1q and induced and bound multiple activation products of complement C1, whereas this was not the case for silica [6], and for CPP and basic calcium phosphate crystals [7].

Coating MSU crystals with serum or plasma markedly reduces the capacity to activate neutrophils [8]. We discovered that the negatively charged MSU crystal surface avidly binds positively charged low density lipoprotein (LDL), mediated via apolipoprotein B (apoB) [8,9]. These huge molecules act much like the non-stick surface of a frying pan, preventing cells from engaging the highly reactive MSU crystal surface [10]. Our collective work revealed MSU crystal-bound LDL, apoB, and later apoE [11] to be among putative promoters of inflammatory quiescence of tissue MSU crystal deposits. Lipoprotein biologist Linda Curtiss was a prized co-mentor in this work, and in manuscripts and grant support that galvanized my permanent move in 1985 to the UC San Diego Division of Rheumatology, Autoimmunity, and Inflammation. I was hired and inspired by Nathan Zvaifler, a towering physician-scientist who was among the pioneers of the modern era of rheumatology [12].

## 5. Major Mediators of Crystal Arthropathy Also Impact Comorbidities

From the early 1980s through the next four decades, major investigative attention was devoted to molecular identification of the cytokines driving gouty inflammation. The early work of Steve Malawista, George Nuki, and colleagues on IL-1β [13] progressed to discovery of MSU crystal-induced NLRP3 inflammasome activation by Jurg Tschopp’s group in 2006 [14]. Our collaborative group identified IL-6 induction by MSU, CPPD, and hydroxyapatite crystals [15]. Over three decades later, IL-6 emerged as a compelling therapy target for acute and chronic arthritis associated with CPPD [16], and potentially as an independent target in gouty arthritis [17], though randomized, controlled trials are awaited. In the 1990s, my group discovered induction of the chemokines IL-8 (CXCL8) and GROα (CXCL1) by MSU crystals, and the profound role in gouty inflammation of the promiscuous CXCR2 receptor for CXCL8 and closely related chemokines including CXCL1 [18,19]. In subsequent work, we determined the central role of the complement C5b-9 membrane attack complex (MAC) in induction of not only tissue CXCL8 in response to MSU crystal injection in vivo, but also neutrophil recruitment in experimental gouty inflammation [20]. We additionally identified induction of CXCL8 in peripheral blood monocytes and monocyte-lineage THP-1 cells by pro-atherogenic oxidized LDL, and major pathogenic roles of CXCL1, CXCL8, and CXCR2 in atherosclerosis in vivo [21–23].

The activities of MAC, other complement proteins, and CXC chemokines as therapy targets in gouty arthritis are now gaining well-justified translational attention [17,24–28]. C5a-generated C5 cleavage on the MSU crystal surface generates C5a that is chemotactic and activating for phagocytes [26], and C5a is a primer of NLRP3 inflammasome activation [27]. MSU crystal-bound C-Reactive Protein (CRP) and natural IgM are now known to substantially promote complement activation and generation of C4a, C3a, and C5a [24,25]. CRP mediates fixation of the complement components C1q, C1r, C1s, and Mannan-binding lectin serine protease 1 (MASP1) to the MSU crystal surface [25]. Moreover, MSU crystals do not show robust complement activation in serum depleted of IgM and CRP [24,25]. Significantly, an inflammatory chemokine loop involving ENA-78/ CXCL5 and neuronal CXCR2 at the dorsal root ganglion was recently found to be a central driver of the unique and characteristically excruciating pain of acute gouty inflammation [28].

CXCL8 is now known to be induced by the tumor necrosis factor superfamily 14 (TNFSF14, formerly known as LIGHT), which is markedly increased in acute gout flare and central to cytokine production and inflammation in that process [17]. In addition, my group, with the lab of David Gonzalez, employed proteomics to discover CXCL8, IL-1β, IL-6, and C5 in a serum protein interactome network altered by treat to target xanthine oxidase inhibitor urate lowering [29]. Collective works by others have heightened relevance of CXCL8 and CXCR2, and not simply other cytokines such as IL-1β and IL-6, to systemic inflammation that modulates atherosclerosis and hypertension [30–33]. In this light, recent gout diagnosis and gout flare are associated with increased cardiovascular events that include deep venous thrombosis, myocardial infarction, and stroke [34–37]. Similarly, acute CPP crystal arthritis flare is significantly associated with increased short and long-term risks for cardiovascular events [38]. Furthermore, based on clinical trial evidence [39], the conventional crystal arthritis flare prophylaxis and treatment drug colchicine was recently FDA-approved for secondary prophylaxis of acute coronary syndrome, superimposed on “preventative standard of care” statin and low dose aspirin.

## 6. Signal Transduction and Crystal Arthritis

Studying MSU crystal-induced pro-inflammatory signal transduction in neutrophils, we dissected the linkages between G protein-dependent and -independent effects, phospholipid remodeling, cytosolic calcium mobilization, and specific protein kinases, [40–44]. Findings that broke new ground included that MSU crystals did not need to be ingested to induce G protein-independent inflammatory signaling, “phospholipid remodeling”, and mobilization of intracellular calcium stores and consequent abrupt inflammatory responses that did not require *de novo* transcription. Furthermore, we provided the first description that MSU and CPP crystals activated NF-κB, c-Jun N-terminal kinase (JNK), other Mitogen Activated Protein Kinase signaling pathways, and the transcription factor AP-1 [45].

### 6.1. Macrophage JNK-AP-1 Pathway Bifurcation from the Lysosomal Program in Crystal-Induced Inflammation

The collaborative team of Isidoro Cobo, Monica Guma, and Chris Glass, with myself and others, recently discovered that MSU crystals induce a metabolic-inflammatory transcriptional program in unprimed murine macrophages diverging markedly from the LPS-induced program via unique upregulation in lipid and amino acid metabolism, glycolysis, and SLC (Solute Carrier) transporter genes [46]. This unique metabolic rewiring of the MSU crystal gene program upregulation is detectable in acute gouty inflammation, and modulated by JNK signaling in a specific, pharmacologically treatable manner, independent of p38 Mitogen Activated Protein Kinase and NLRP3 inflammasome activation [46]. The consortium next determined that macrophage responses to MSU, CPPD crystals, and silica particles require an AMP Activated Protein Kinase (AMPK)-dependent transcriptional network for lysosomal acidification [47]. The critical players for induction of lysosomal acidification genes were the lysosomal biogenesis regulatory transcription factors (TFs) TFEB and TFE3, and the epigenetic regulators DNA methyl transferase 3a (DNMT3A) and Disruptor of Telomeric Silencing 1-Like (DOT1L) histone methyltransferase axis [47]. A bifurcating JNK-AP-1 pathway was shown to independently drive cytokine and chemokine expression to inflammatory crystals and particulates [47].

### 6.2. AMPK Effects Are Double-Edged in Crystal Arthropathy

Perhaps nothing illustrates the value of “following the molecule” more than tracking the double-edged effects of AMPK in crystal-induced inflammation. AMPK, whose primary function is nutritional biosensing, exerts substantial anti-inflammatory effects that suppress crystal-induced inflammation, including inhibition of NF-κB activation, induction of Sirtuin 1 activation, modulation of macrophage polarization [48], and the promotion and support of autophagy [49–52]. Notably, AMPK activates the enzymatic protein degradative machinery of the autophagosome using the aforementioned transcriptional network for pro-inflammatory lysosomal biogenesis and acidification [47].

Ru Bryan spearheaded our seminal efforts to examine the potential therapeutic role of AMPK activation in gout [48] and other diseases mediated by inflammatory processes [53,54]. Key findings in gout included that macrophage AMPK is inhibited by incubation with MSU crystals, where AMPK is activated by low dose colchicine [48]. AMPK activity transduces colchicine anti-inflammatory effects in macrophages, inhibition of the NLRP3 inflammasome, release of multiple inflammatory cytokines, and polarization of macrophages to anti-inflammatory M2 differentiation [48]. We similarly showed AMPK to transduce anti-inflammatory effects in gout of the peroxisome proliferator-activated receptor partial agonist arhalofenate [48a]. Murine AMPK knockout markedly increases the inflammatory response to MSU crystals in vivo, whereas pharmacologic AMPK activation suppresses the response [48]. More recently, other investigators uncovered association of prescription of the AMPK activator metformin with less incident gout [55]. In some, but not all retrospective studies, a lower frequency of gout flares was seen in metformin recipients [49,56]. However, retrospective association studies in populations with metabolic syndrome and type 2 diabetes are subject to limitations via confounders such as polypharmacy, not limited to use of SGLT2 inhibitors with anti-inflammatory activity [57,58].

### 6.3. Phagocyte Membrane Proteins Central to Crystal-Induced Inflammation

Many cell membrane proteins, including CD44 [59] and C-type lectin domain family 12 member A (CLEC12A) [60], engage the highly reactive negatively charged MSU crystal surface, with pro-inflammatory functional consequences that complement the effects of nonspecific membrane perturbation by the crystals. Our seminal work on the MSU crystal-bound cell membrane proteome of phagocytes revealed that TLR2, TLR4, their cytoplasmic adaptor signaling protein Myeloid Differentiation Primary Response 88 (MyD88), and the classical monocyte marker CD14 to be central in gouty inflammation [61,62]. We discovered that MSU crystals bind CD14, and that CD14 knockout macrophages ingest MSU crystals, but with an inflammation-resolving rather than pro-inflammatory response unless the crystals are pre-coated with soluble CD14 [62]. Recent work by Leo Joosten and colleagues has buttressed how CD14 factors in the inflammatory monocyte phenotype and in inflammatory responses to MSU crystals [63]. Also, HLA-DQA1 highCD14 +monocytes are now known to heighten immune responses and bone erosion in advanced gouty arthropathy.

### 6.4. The DNA Methylome and Epigenetic Training of Gouty Inflammation

Working with Wei Wang and Tony Merriman, we were the first to characterize the DNA methylome of patients with gout [64]. We studied peripheral blood mononuclear cells from my own UC San Diego gout cohort and controls, with Dr. Merriman’s New Zealand cohort for validation [64]. We discovered a distinct fingerprint of differentially methylated loci in gout. For example, the IL-23 receptor gene *IL23R*, a mediator of granuloma formation and cell invasiveness, was among the differentially methylated risk genes for gout. DNA methylome differences that distinguished gout from non-gout control subjects also were found in pathways of cell homeostasis, adaptive immunity, and inflammation, including signaling of B cell and T cell receptors, IL-17 and Th17 cells, AMPK, longevity regulation, autophagy, NF-κB, and multiple other transcriptional networks [64]. Also, the circadian entrainment pathway was differentially methylated in patients with gout [64], highly relevant to the nocturnal flare onset characteristic of gout. Differentially DNA methylated transcriptional network pathways and regulatory factors for osteoclastogenesis were also associated with gout.

Now, multiple lines of evidence highlight the impact of epigenetic regulation of gouty inflammation and susceptibility to developing gout, via clonal hematopoiesis of indeterminate potential (CHIP) and the epigenetic training of innate immune memory in hyperuricemia and gout illuminated by the Joosten group [65–69]. Our DNA methylome data also buttress more recent evidence for adaptive immunity as a key factor regulating the inflammatory response to MSU crystals and the unique biology of tophaceous, bone erosive gout pathology with potentiation of osteoclastogenesis, including at the tophus-bone interface [70–72].

## 7. Lubricin and Lessons Learned from Erosive Gout Without Hyperuricemia

Recent work from my lab, with a large global network of collaborators including Khaled Elsaid, Tony Merriman, Jack Karsh, and David Gonzalez, illuminated unique, hyperuricemia-autonomous aspects in development of gouty arthritis and progression to erosive tophaceous joint disease [73]. We studied a 22-year-old female who developed erosive gout with intra-articular tophi, destructive gouty arthritis of the hip that led to total joint arthroplasty, and stereotypical gout flares. The proband had a normal serum urate of 5 mg/dL [73], and gout flares eventually improved on allopurinol that lowered her serum urate to 2 mg/dL. Kindred members were also studied. There was no family history or common comorbidity of gout, but the proband went on to develop Crohn’s disease in her mid-thirties. Genome-wide sequencing revealed a proband inflammatory diathesis, which included multiple NPRP3 gain of function gene variants. Using proteomics and other confirmatory assays, we observed attenuation of circulating lubricin in the proband, mediated by an increase in activity of lubricin-cleaving proteases such as Cathepsin G and likely neutrophil elastase as well, and linked with deficient inhibition of lubricin proteolysis by thrombospondin and the serine protease inhibitor SERPINB6 [73]. Serum lubricin was also very low in a substantial subset of gout patients. Novel in vitro and in vivo studies revealed that lubricin, at concentrations present in normal synovial fluid, markedly inhibits crystallization of MSU. Lubricin was also shown to suppress the ability of activated macrophages to express xanthine oxidase and urate generation [73].

Collective results uncovered a highly consequential lubricin mechanistic circuit within the joint space, vulnerable to IL-1β, to TLR2-mediated synovial fibroblast lining cell signaling, and to increased activity of lubricin degrading proteases [73,74]. Elsaid and colleagues laid the groundwork by finding that lubricin not only inhibits phagocytosis of MSU crystals via engaging CD44 but also limits MSU crystal-induced inflammation in vivo [59]. We discovered lubricin to restrain not only intra-articular generation and crystallization of urate, but also macrophage xanthine oxidase and urate release.

Lubricin suppresses synovitis and also coats and lubricates the cartilage surface (where MSU crystals avidly deposit) [74]. Since joint fluid lubricin levels drop in association with inflammation in chronic joint disease [74], dysregulated homeostasis of lubricin has the capacity to diminish natural limitations on development of gout, flares, and erosive tophaceous joint disease. Articular lubricin deficiency can thereby contribute to the strong association of gout with osteoarthritis of the first metatarsophalangeal and other joints [74].

## 8. PP_i_ Metabolism in CPPD, Other Forms of Ectopic Calcification, and Physiology

I have long worked to address unmet needs in CPPD, where dysregulation of relevant nucleotide and PP_i_ biology remains under-investigated [75]. Having followed Dan McCarty’s groundbreaking work on ATP metabolism and PP_i_ biology in CPPD, I aimed to molecularly identify etiologic players and pathways. Dysregulated PP_i_ metabolism is fundamental to the development of CPPD [75,76]. Starting in the early 1990s, we seminally identified the large, homodimeric transmembrane ecto-enzyme plasma cell membrane glycoprotein-1 (PC-1) as the principal extracellular PP_i_-generating ATP-hydrolyzing nucleoside triphosphate pyrophosphohydrolase (NPP) in chondrocytes and osteoblasts, including at sites of calcification [77,78]. We observed that PC-1, encoded by the *ENPP1* gene and ultimately renamed NPP1, is increased in articular cartilage in degenerative joint disease and at loci of chondrocyte hypertrophy and calcification [79,80].

**Figure 3.**
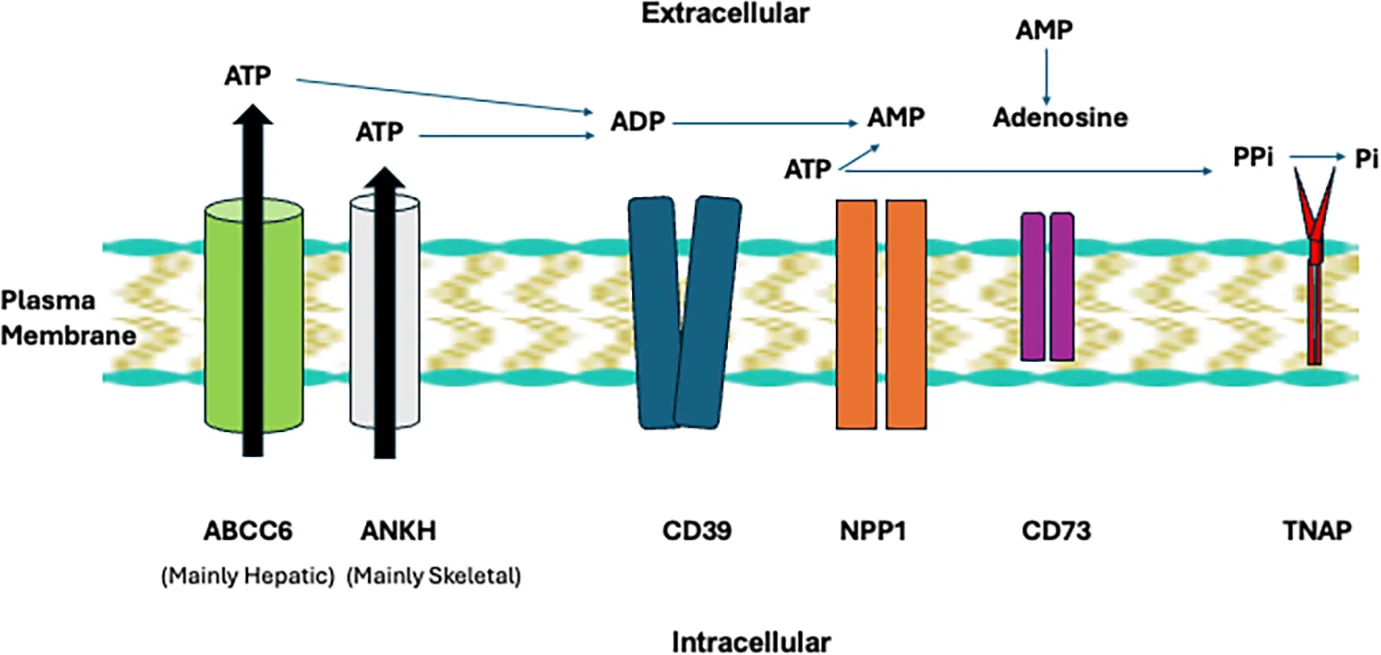
NPP1 and TNAP in the plasma membrane enzyme and transporter chain that regulates extracellular ATP and generation of AMP, adenosine, PP_i_, and inorganic phosphate (P_i_). The broad physiological functions of this chain are discussed in the text. Image of plasma membrane via Good Free Photos https://www.goodfreephotos.com/.

Our work seminally illuminated NPP1 physiology in a plasma membrane enzyme and transporter chain that regulates extracellular ATP, AMP and adenosine, PP_i_, and inorganic phosphate (P_i_) (Figure 3) [81–87]. NPP1 is upstream in the chain containing the ATP excretory transporters ANKH (in skeletal tissues) and ABCC6 in the liver, the ecto-nucleoside triphosphate diphosphohydrolase-1 CD39 that competes with NPP1 to generate AMP, CD73 that catalyzes adenosine from AMP generated in part by NPP1, and tissue nonspecific alkaline phosphatase (TNAP) that hydrolyzes PP_i_ to generate P_i_ [87–89]. We found NPP1 to be essential for generation of PP_i_ in bone and cartilage via what is now recognized to be hydrolysis of ATP released via ANKH [86]. We discovered that excess of not only ANKH but also extracellular PP_i_ alone promote cartilage degeneration by inducing MMP-13 [87], confirmed independently later [90]. Also, we uncovered that a subset of what had been labeled “idiopathic/sporadic CPPD” was heritably influenced via a −4-bp G-to-A *ANKH 5′*-untranslated region polymorphism that upregulates ANKH expression and extracellular PP_i_ generation *in vitro* [91]. This genetic association was later confirmed by the Abhishek group, who revealed it to be independent of age and osteoarthritis [92].

Significantly, we now know that extracellular citrate accumulation factors into certain aging-related pathologies. Including osteoarthritis [93,94] and that ANKH exports citrate [95,96]. This ANKH function could promote the pro-inflammatory senescence associated secretory phenotype, findings noteworthy because of recent linkage of articular chondrocyte senescence to CPP crystal deposition [97]. In addition, the seminal finding linking familial CPPD to childhood onset seizures now may well be explained by ANKH transport of citrate [98].

Intrigued by the fundamental regulation of chondrocyte extracellular PP_i_ by specific growth factors, exemplified by mutually antagonistic stimulatory and inhibitory effects of TGFβ and IGF-I, respectively [99], we investigated the effects of Bone Morphogenetic Proteins, PTHrP, and bFGF [100,101]. We also helped determine not only that the TGFβ effect to promote PP_i_ release is heightened with donor age in human articular chondrocytes [102], but also that IL-1β suppresses chondrocyte and calcifying chondrocyte-derived apoptotic body NPP1 and PP_i_ levels [103,104]. Establishing marked dominance of NPP1 as the articular chondrocyte PP_i_ generating enzyme [105], we performed requisite molecular studies proving that the two Cartilage Intermediate Layer Protein (CILP) isoforms are not NPP enzymes. Instead, we found that CILP-1 indirectly regulates chondrocyte PP_i_ levels by modulating responsiveness to IGF-I [106].

Tony Merriman’s groundbreaking genome-wide association study (GWAS) on African and European chondrocalcinosis cohorts has implicated an *ENPP1* variant associated with increased NPP1 tissue expression [107]. NPP1 inhibitory nucleotide analog treatment suppresses ATP-induced CPP crystal deposition by chondrocytes *in vitro*, which buttresses NPP1 translational impact for CPPD [108]. However, we uncovered deleterious effects of even partial NPP1 deficiency that impose challenges to development of NPP1 inhibition therapeutics for CPPD.

### 8.1. NPP1 Physiology Highlighted by Broad Disease States in NPP1 and PP_i_ Deficiency States

In physiology, the major roles of PP_i_ are suppression of ectopic calcification with hydroxyapatite and other basic calcium phosphate crystals, and regulation of skeletal calcification, partly by providing PP_i_ for P_i_ generation by TNAP [83]. Moreover, the NPP1-PP_i_ -CD73-adenosine axis (Figure 3) contributes broadly to tissue homeostasis, including in the vasculature, bone, and spinal ligaments [109].

With Jose Luis Millan, we identified the intimate relationship in physiologic bone calcification between NPP1 and PP_i_-hydrolyzing (TNAP), mediated in large part via osteoblast matrix vesicles, and the calcification suppressor osteopontin, which is induced by both PP_i_ and P_i_ [82–85]. This work was foundational to later elucidation that NPP1 deficiency promotes not only osteomalacia (Autosomal Recessive Hypophosphatemic Rickets type 2 (ARHR2)), but also early-onset osteoporosis [109].

Our key translational achievement in NPP1-related tissue homeostasis and calcification biology stemmed from a study in the early 1980s of an infant from a consanguineous marriage, who developed marked arterial stenosis mediated by pronounced neointimal hyperplasia and arterial calcification, hypertension, heart failure, and periarticular calcification [110,111]. This rare phenotype, little known in 1980, is now termed generalized arterial calcification of infancy (GACI) [109,112,113]. Pediatrician Frank Rutsch noted very low urinary PP_i_ in the infant {110], and moved to my lab for postdoctoral training, launching the first studies to molecularly define pathogenesis, clinical course, and the GACI diagnostic *ENPP1* mutational spectrum that remains in use since then [110–114]. Carrying huge mortality in year 1 of life, GACI evolves to hypophosphatemic rickets in childhood persisting into adulthood [109,111,113]. We seminally ascertained that severe NPP1 deficiency in GACI causes near total absence of circulating PP_i_ [111], a critical biomarker in current pivotal clinical trials on Fc-conjugated NPP1 for GACI.

Pseudoxanthoma elasticum (PXE), a childhood-onset disease associated with systemically low PP_i_ and ectopic calcification, is primarily due to deficiency of the hepatocyte ATP-excreting transporter ABCC6 [Figure 3] [88,109]. Frank Rutsch and I discovered that a small subset of PXE is caused by NPP1 deficiency, and conversely that a small subset of ABCC6-deficient individuals presents with GACI rather than PXE [88]. Later, my group discovered that NPP1 physiologically suppresses ectopic calcification in large part by limiting ectopic chondrogenesis [114]. Similarly, we found that ANKH limits ectopic chondrogenesis, acting at the mesenchymal stem cell level and mediated by markedly increased Vanin-1 pantetheinase [115], which we discovered to transduce neointimal hyperplasia [116]. We now know that human NPP1 deficiency states can cause other diseases of heterotopic ossification, such as Ossification of the Posterior Longitudinal Ligament (OPLL) and Diffuse Idiopathic Skeletal Hyperostosis (DISH), and that ANKH deficiency can cause progressive craniofacial bone hyperostosis [109].

Clearly, therapeutic opportunities for boosting NPP1, PP_i_, and downstream adenosine are expansive and feasible [83,109]. For example, my group found that even *ENPP1* haploinsufficiency promotes murine post-injury arterial stenosis via neointimal hyperplasia, and that arterial obstruction happens in the matter of a few weeks after injury, without lesion calcification in that time frame [117]. Frank Rutsch and colleagues later showed the protective effect of NPP1 on murine post-injury carotid artery neointimal hyperplasia to be mechanistically adenosine-dependent and prevented in vivo by Fc-conjugated NPP1 administration [118,119].

### 8.2. The NPP1-PP_i_-CD73-Adenosine Axis in Osteoarthritis

Is synovial joint homeostasis of potential area for therapeutic modulation within the NPP1-PP_i_ -CD73-adenosine axis? In this context, increased chondrocyte hypertrophic differentiation exerts fundamental pro-calcifying effects in osteoarthritis and CPPD joint cartilages [120,121]. Chondrocyte hypertrophy is a physiologic process in growth plate mineralization, associated with vascular invasion, matrix vesicle shedding, and basic calcium phosphate crystal deposition [120,121]. There are over a dozen mediators involved in the growth plate process, with the Wnt/β-catenin pathway a prime example, and some of these mediators contribute to osteoarthritis pathophysiology in articular cartilage [120,121]. Yingzi Yang, with my collaborative assistance, discovered that NPP1 suppresses chondrocyte hypertrophy and ectopic calcification in part by inhibiting Hedgehog signaling, activating Gα_s_-PKA signaling, and thereby supporting synovial joint homeostasis [122]. That said, the notion of augmenting the large molecule NPP1 in the joint, and thereby augmenting PP_i_, risks more pathologic calcification in osteoarthritis. A more pragmatic approach has been taken by Bruce Cronstein and colleagues. They showed chondroprotective potential of liposomal adenosine, and that small molecule activation of the chondrocyte adenosine A2A receptor limits chondrocyte senescence, mitochondrial damage, pathways driving extracellular matrix damage, and experimental osteoarthritis [123–127].

## 9. Ectopic Calcification in the Setting of Cartilage “Inflamm-Aging” in Osteoarthritis

Our studies on cartilage “inflamm-aging” in osteoarthritis and associated pathologic cartilage calcification identified chronic, “low-grade inflammation in cartilage” and associated dysregulation of proteostasis and mitochondrial function integral to articular cartilage aging and pro-mineralizing chondrocyte hypertrophy. Specifically, our seminal work showed dysregulation of the unfolded protein response and ubiquitin proteasome system in chondrocytes in cartilage injury and osteoarthritis, and how decreased AMPK activity intersects with defective proteostasis [128–131]. We also demonstrated activation of AMPK to be deficient in human knee osteoarthritis chondrocytes, and that IL-1β was one of the inflammatory stimuli that reduce chondrocyte AMPK activity [132].

A low ratio of ATP to AMP promotes AMPK activation, and mitochondrial oxidative phosphorylation is a substantial generator of ATP in chondrocytes. We discovered that pig knee chondrocytes of aged Hartley guinea pigs, which develop OA spontaneously, had “ATP hunger” due to mitochondrial dysfunction, with evidence for decreased oxidative phosphorylation [133], an effect we linked to increased chondrocyte nitric oxide [134]. The guinea pig chondrocytes developed increased PP_i_ output and increased NPP activity as OA progressed with increasing age [133]. We also elucidated AMPK chondroprotective effects by augmenting impaired mitochondrial biogenesis in osteoarthritic cartilage, and preservation of mitochondrial DNA integrity and function [135,136]. These collective findings seminally drove the concept that chondrocyte mitochondrial dysfunction factors into the linkage of osteoarthritis with CPPD in aging.

Our pivotal preclinical studies established that AMPK activators such as metformin limit experimental knee OA [54,137]. Though clinical trial results have been variable [138], we now know that metformin use in type 2 diabetics has been linked to decreased risk of both new-onset OA and/or eventual total hip or knee joint replacement [54,139–143]. Importantly, the past literature indicates that coexistent CPPD is not necessarily an anatomic progression signal for osteoarthritis [144,145]. Hence, the upregulated intracellular NPP1-AMP+PP_i_ and extracellular NPP1-PP_i_-CD73-adenosine pathways in aging and OA cartilages may well be an adaptive, protective mechanism for OA progression by hydrolyzing ATP to generate intracellular AMP for activation of AMPK and producing extracellular PP_i_ to suppress basic calcium phosphate crystal formation. However, the cost is promotion of CPP crystal formation under suitable conditions in the extracellular matrix.

### Chondrocyte Hypertrophy Driven by an Inflamm-Aging Network

In formative studies, we laid out a network of inflamm-aging mediators that promotes chondrocyte hypertrophy in osteoarthritic cartilage [146–158]. In this context, with Martin Lotz, we first implicated chemokines, particularly CXCL1 and CXCL8, which share signaling via CXCR2, to promote articular chondrocyte hypertrophy. Recent work by others, uncovering inflammatory and pre-hypertrophic cell populations in human knee OA via multi- omics data integration, revealed CXCL8 and CXCR2 enrichment specifically in the “inflammatory chondrocyte” subset [159]. In addition, cartilage homeostatic functions of CXCR2 via heparin-bonding chemokines were recently uncovered [160].

The chondrocyte hypertrophy inflamm-aging network, that we discovered largely over a decade, included externalization of Transglutaminase 2 (TG2) bound to GDP, transamidation of specific S100 calgranulins, release of the damage associated molecular pattern (DAMP) High Mobility Group Box 1(HMGB1), and signaling by Toll-like Receptors (TLRs), CD36, and the receptor for advanced glycation products (RAGE) [149–158]. The dual enzyme TG2 acts in a calcium-dependent manner to crosslink and alter folding in proteins by catalyzing isopeptide bond formation between γ-carboxamide groups of glutamine side chains and ε-amino groups of lysine side chains. Transamidation and deamidation reactions of TG2 are silenced under low calcium conditions that favor GTP binding to TG2, conformational change in the enzyme, and hydrolysis of GTP to GDP that confers the GTPase signal transduction activity we determined to mediate a distinct pathway for chondrocyte hypertrophy [153,154]. Notably, we found that IL-1β induces pro-calcifying activity TG2 and a second articular chondrocyte transglutaminase Factor XIIIA [157]. Following TG2 to the process of artery calcification, we discovered a key role of TG2 in the calcifying differentiation and activity of arterial smooth muscle cells [161].

TG2 is now an emerging target for fibrotic diseases mediated by extracellular matrix protein crosslinking, exemplified by scleroderma. However, we followed TG2 to relevant physiology and established TG2 to mediate how macrophages take up apoptotic neutrophils in the joint (efferocytosis) [162] and promote the resolution phase of gouty inflammation via innate immunity reprogramming that includes more TGFβ release [163]. Our work also revealed that macrophage TG2 limits both atherosclerosis [164] and post-injury neointimal hyperplasia [165], pointing to further challenges to developing pharmacologic TG2 antagonism.

## 10. Concluding Perspectives

My basic-translational research has followed many target molecules (eg, apoB, C1 activation, C5b-9, CXCL8, CXCR2, NPP1, TG2, AMPK, lubricin) in what is now appreciated to be the tightly knit space spanning crystal disease and specific comorbidities. The work has used big data approaches to decipher disease, and has developed clinical and diagnostic biomarkers and emerging therapeutics. A prime example of the new therapeutics is NPP1 conjugated to Fc, which is in advanced clinical trials for GACI and PXE and in early development for calciphylaxis and other diseases of heterotopic calcification and ossification. Another example is clinical trials of the AMPK activator metformin in osteoarthritis.

I continue to marvel at how and where the road I have traveled on continues to be carved forward. When I started gout research in 1981, neutrophils, phagocytosis, complement, chemotaxis, and the MSU crystal-bound proteome were at the leading edge of initiation and quiescence of gouty inflammation. Over four decades later, macrophage polarization and cytokines, NLRP3, signal transduction and transcription activation, AMPK, autophagy, lubricin, the lysosomal biogenesis and activation gene program, and CHIP and the epigenome in training innate immunity and adaptive immunity [64,70,71] are major talking points. Remarkably, much of the conversation has circled back to neutrophils, since efferocytosis of apoptotic neutrophils by macrophages [162,163] and neutrophil extracellular trap formation (NETosis) [166–168] are factors in quiescence of gouty inflammation and tophus formation.

Commencing work on CPPD and pathologic calcification over three decades ago, it would have been far-fetched to imagine that the enzyme, in excess, driving chondrocyte extracellular PP_i_ production would also be central, when deficient, in arterial media calcification and intimal hyperplasia. The same is the case for the CXCR2 ligand chemokines axis, integral to crystal-induced inflammation, being involved in articular chondrocyte hypertrophy and atherosclerosis while maintaining chondrocyte homeostasis; likewise for the broad pathologic and homeostasis effects of TG2 in cartilage and arterial calcification, atherosclerosis, intimal hyperplasia, and resolution of MSU crystal-associated inflammation. But the lesson is that more can always be learned, through ongoing investigation and the pursuit of more perfect enlightenments. Quoting Linus Pauling: “If you want to have good ideas, you must have many ideas”, and as Albert Szent-Györgyi said, “Research is to see what everybody else has seen, and to think what nobody else has thought.”

## Abbreviations

ABCC6: ATP-Binding Cassette Sub-Family C Member 6
AMPK: AMP-Activated Protein Kinase
ARHR2: Autosomal Recessive Hypophosphatemic Rickets Type 2
CHIP: Clonal Hematopoiesis of Indeterminate Potential
CILP: Cartilage Intermediate Layer Protein
CLEC12A: C-Type Lectin Domain Family 12 Member A
CPPD: Calcium Pyrophosphate Deposition Disease
CRP: C-Reactive Protein
CXCL: C-X-C Chemokine Ligand
CXCR: C-X-C Chemokine Receptor
DAMP: Damage-Associated Molecular Pattern
DISH: Diffuse Idiopathic Skeletal Hyperostosis
DNMT3A: DNA Methyl Transferase 3a
DOTL1: Disruptor of Telomeric Silencing 1-Like
ENA-78: Epithelial-Derived Neutrophil Activating Peptide 78/CXCL5
GACI: Generalized Arterial Calcification of Infancy
HMGB1: High Mobility Group Box 1
IGF: Insulin Like Growth Factor
IL: Interleukin
JNK: Jun N-Terminal Kinase
MAC: Membrane Attack Complex
MASP1: Mannan-Binding Lectin Serine Protease 1
MMP: Matrix Metalloproteinase
MSU: Monosodium Urate
MyD88: Myeloid Differentiation Primary Response 88
NLRP3: NOD-, LRR- and Pyrin Domain-Containing Protein)
NPP1: Ectonucleotide Pyrophosphatase/Phosphodiesterase 1
OPLL: Ossification of the Posterior Longitudinal Ligament
PXE: Pseudoxanthoma Elasticum
SLC: Solute Carrier
RAGE: Receptor for Advanced Glycation Products
TF: Transcription Factor
TG2: Transglutaminase 2
TGF: Transforming Growth Factor
TLR: Toll-Like Receptor
TNAP: Tissue Nonspecific Alkaline Phosphatase

## References

[1] Giclas PC; Ginsberg MH; Cooper NR Immunoglobulin G independent activation of the classical complement pathway by monosodium urate crystals. J. Clin. Investig. 1979, 63, 759–764. 10.1172/JCI109360. 438335 PMC372012

[2] Ginsberg MH; Jaques B; Cochrane CG; Griffin JH Urate crystal—Dependent cleavage of Hageman factor in human plasma and synovial fluid. J. Lab. Clin. Med. 1980, 95, 497–506. 6898641

[3] Spilberg I; Rosenberg D; Mandell B Induction of arthritis by purified cell-derived chemotactic factor: Role of chemotaxis and vascular permeability. J. Clin. Investig. 1977, 59, 582–585. 10.1172/JCI108674. 838864 PMC333396

[4] Ginsberg MH; Kozin F Mechanisms of cellular interaction with monosodium urate crystals. IgG-dependent and IgG-independent platelet stimulation by urate crystals. Arthritis Rheum. 1978, 21, 896–903. 10.1002/art.1780210805. 737013

[5] Terkeltaub R Physiologic and pathologic functions of the NPP nucleotide pyrophosphatase/phosphodiesterase family focusing on NPP1 in calcification. Purinergic Signal. 2006, 2, 371–377. 10.1007/s11302-005-5304-3. 18404477 PMC2254483

[6] Terkeltaub R; Tenner AJ; Kozin F; Ginsberg MH Plasma protein binding by monosodium urate crystals. Analysis by two-dimensional gel electrophoresis. Arthritis Rheum. 1983, 26, 775–783. 10.1002/art.1780260612. 6305372

[7] Terkeltaub RA; Santoro DA; Mandel G; Mandel N Serum and plasma inhibit neutrophil stimulation by hydroxyapatite crystals. Evidence that serum alpha 2-HS glycoprotein is a potent and specific crystal-bound inhibitor. Arthritis Rheum. 1988, 31, 1081–1089. 10.1002/art.1780310901. 2844196

[8] Terkeltaub R; Martin J; Curtiss LK; Ginsberg MH Apolipoprotein B mediates the capacity of low density lipoprotein to suppress neutrophil stimulation by particulates. J. Biol. Chem. 1986, 261, 15662–15667. 3096995

[9] Terkeltaub R; Curtiss LK; Tenner AJ; Ginsberg MH Lipoproteins containing apoprotein B are a major regulator of neutrophil responses to monosodium urate crystals. J. Clin. Investig. 1984, 73, 1719–1730. 10.1172/JCI111380. 6725556 PMC437084

[10] Terkeltaub R; Smeltzer D; Curtiss LK; Ginsberg MH Low density lipoprotein inhibits the physical interaction of phlogistic crystals and inflammatory cells. Arthritis Rheum. 1986, 29, 363–370. 10.1002/art.1780290309. 3964313

[11] Terkeltaub RA; Dyer CA; Martin J; Curtiss LK Apolipoprotein (apo) E inhibits the capacity of monosodium urate crystals to stimulate neutrophils. Characterization of intraarticular apo E and demonstration of apo E binding to urate crystals in vivo. J. Clin. Investig. 1991, 87, 20–26. 10.1172/JCI114971. 1985096 PMC294981

[12] Firestein GS; Corr M In memoriam: Nathan, J. Zvaifler, MD, 1927–2015. Arthritis Rheumatol. 2015, 67, 1143. 10.1002/art.39089. 25917709

[13] Di Giovine FS; Malawista SE; Nuki G; Duff GW Interleukin 1 (IL 1) as a mediator of crystal arthritis. Stimulation of T cell and synovial fibroblast mitogenesis by urate crystal-induced IL 1. J. Immunol. 1987, 138, 3213–3218. 3033070

[14] Martinon F; Pétrilli V; Mayor A; Tardivel A; Tschopp J Gout-associated uric acid crystals activate the NALP3 inflammasome. Nature 2006, 440, 237–241. 10.1038/nature04516. 16407889

[15] Guerne PA; Terkeltaub R; Zuraw B; Lotz M Inflammatory microcrystals stimulate interleukin-6 production and secretion by human monocytes and synoviocytes. Arthritis Rheum. 1989, 32, 1443–1452. 10.1002/anr.1780321114. 2554932

[16] Carrabin S; Houze M; Jauffret C; Bardin T; Ea H; Lioté F; Richette P; Pascart T; Latourte A Efficacy and Safety of Tocilizumab in the Treatment of Chronic Inflammatory Forms of CPPD: Retrospective Study of 55 Cases [abstract]. Arthritis Rheumatol. 2024, 76 (Suppl. 9). Available online: https://acrabstracts.org/abstract/efficacy-and-safety-of-tocilizumab-in-the-treatment-of-chronic-inflammatory-forms-of-cppd-retrospective-study-of-55-cases/ (accessed on 13 June 2025).

[17] Ea HK; Kischkel B; Chirayath TW; Klück V; Aparicio C; Loeung HU; Manivet P; Jansen T; Zarka M; Lioté F; Systemic inflammatory cytokine profiles in patients with gout during flare, intercritical and treat-to-target phases: TNFSF14 as new biomarker. Ann. Rheum. Dis. 2024, 83, 945–956. 10.1136/ard-2023-225305. 38373842

[18] Terkeltaub R; Zachariae C; Santoro D; Martin J; Peveri P; Matsushima K Monocyte-derived neutrophil chemotactic factor/interleukin-8 is a potential mediator of crystal-induced inflammation. Arthritis Rheum. 1991, 34, 894–903. 10.1002/art.1780340716. 2059236

[19] Terkeltaub R; Baird S; Sears P; Santiago R; Boisvert W The murine homolog of the interleukin-8 receptor CXCR-2 is essential for the occurrence of neutrophilic inflammation in the air pouch model of acute urate crystal-induced gouty synovitis. Arthritis Rheum. 1998, 41, 900–909. 10.1002/1529-0131(199805)41:5<900::AID-ART18>3.0.CO;2-K. 9588743

[20] Tramontini N; Huber C; Ru L-B; Terkeltaub RA; Kilgore KS Central role of complement membrane attack complex in monosodium urate crystal-induced neutrophilic rabbit knee synovitis. Arthritis Rheum. 2004, 50, 2633–2639. 10.1002/art.20386. 15334478

[21] Terkeltaub R; Banka CL; Solan J; Santoro D; Brand K; Curtiss LK Oxidized LDL induces monocytic cell expression of interleukin-8, a chemokine with T-lymphocyte chemotactic activity. Arterioscler. Thromb. 1994, 14, 47–53. 10.1161/01.atv.14.1.47. 8274477

[22] Boisvert WA; Santiago R; Curtiss LK; Terkeltaub RA A leukocyte homologue of the receptor CXCR-2 mediates the accumulation of macrophages in atherosclerotic lesions of LDL receptor-deficient mice. J. Clin. Investig. 1998, 101, 353–363. 10.1172/JCI1195. 9435307 PMC508574

[23] Boisvert WA; Rose DM; Johnson KA; Fuentes ME; Lira SA; Curtiss LK; Terkeltaub RA Up-regulated expression of the CXCR2 ligand KC/GRO-alpha in atherosclerotic lesions plays a central role in macrophage accumulation and lesion progression. Am. J. Pathol. 2006, 168, 1385–1395. 10.2353/ajpath.2006.040748. 16565511 PMC1606562

[24] Wessig AK; Hoffmeister L; Klingberg A; Alberts A; Pich A; Brand K; Witte T; Neumann K Natural antibodies and CRP drive anaphylatoxin production by urate crystals. Sci. Rep. 2022, 12, 4483. 10.1038/s41598-022-08311-z. 35296708 PMC8924570

[25] Alberts A; Klingberg A; Wessig AK; Combes C; Witte T; Brand K; Pich A; Neumann K C-reactive protein (CRP) recognizes uric acid crystals and recruits proteases C1 and MASP1. Sci. Rep. 2020, 10, 6391. 10.1038/s41598-020-63318-8. 32286427 PMC7156728

[26] Russell IJ; Mansen C; Kolb LM; Kolb WP Activation of the fifth component of human complement (C5) induced by monosodium urate crystals: C5 convertase assembly on the crystal surface. Clin. Immunol. Immunopathol. 1982, 24, 239–250. 10.1016/0090-1229(82)90235-5. 6749358

[27] Brandstetter C; Holz FG; Krohne TU Complement Component C5a Primes Retinal Pigment Epithelial Cells for Inflammasome Activation by Lipofuscin-mediated Photooxidative Damage. J. Biol. Chem. 2015, 290, 31189–31198. 10.1074/jbc.M115.671180. 26565031 PMC4692241

[28] Yin C; Liu B; Dong Z; Shi S; Peng C; Pan Y; Bi X; Nie H; Zhang Y; Tai Y; CXCL5 activates CXCR2 in nociceptive sensory neurons to drive joint pain and inflammation in experimental gouty arthritis. Nat. Commun. 2024, 15, 3263. 10.1038/s41467-024-47640-7. 38627393 PMC11021482

[29] Sanchez C; Campeau A; Ru L-B; Mikuls TR; O’Dell JR; Gonzalez DJ; Terkeltaub R Effective xanthine oxidase inhibitor urate lowering therapy in gout is linked to an emergent serum protein interactome of complement and inflammation modulators. Sci. Rep. 2024, 14, 24598. 10.1038/s41598-024-74154-5. 39426967 PMC11490615

[30] Kienhorst LB; van Lochem E; Kievit W; Dalbeth N; Merriman ME; Phipps-Green A; Loof A; van Heerde W; Vermeulen S; Stamp LK; Gout Is a Chronic Inflammatory Disease in Which High Levels of Interleukin-8 (CXCL8), Myeloid-Related Protein 8/Myeloid-Related Protein 14 Complex, and an Altered Proteome Are Associated With Diabetes Mellitus and Cardiovascular Disease. Arthritis Rheumatol. 2015, 67, 3303–3313. 10.1002/art.39318. 26248007

[31] Dhayni K; Zibara K; Issa H; Kamel S; Bennis Y Targeting CXCR1 and CXCR2 receptors in cardiovascular diseases. Pharmacol. Ther. 2022, 237, 108257. 10.1016/j.pharmthera.2022.108257. 35908611

[32] Tillmann S; Bernhagen J; Noels H Arrest Functions of the MIF Ligand/Receptor Axes in Atherogenesis. Front. Immunol. 2013, 4, 115. 10.3389/fimmu.2013.00115. 23720662 PMC3655399

[33] Martynowicz H; Janus A; Nowacki D; Mazur G The role of chemokines in hypertension. Adv. Clin. Exp. Med. 2014, 23, 319–325. 10.17219/acem/37123. 24979502

[34] Cipolletta E; Nakafero G; McCormick N; Yokose C; Avery AJ; Mamas MA; Choi HK; Tata LJ; Abhishek A Cardiovascular events in patients with gout initiating urate-lowering therapy with or without colchicine for flare prophylaxis: A retrospective new-user cohort study using linked primary care, hospitalisation, and mortality data. Lancet Rheumatol. 2025, 7, e197–e207. 10.1016/S2665-9913(24)00248-0. 39708831

[35] Cipolletta E; Nakafero G; Richette P; Avery AJ; Mamas MA; Tata LJ; Abhishek A Short-Term Risk of Cardiovascular Events in People Newly Diagnosed with Gout. Arthritis Rheumatol. 2025, 77, 202–211. 10.1002/art.42986. 39279144 PMC11782110

[36] Cipolletta E; Tata LJ; Nakafero G; Avery AJ; Mamas MA; Abhishek A Risk of Venous Thromboembolism with Gout Flares. Arthritis Rheumatol. 2023, 75, 1638–1647. 10.1002/art.42480. 36808284

[37] Cipolletta E; Tata LJ; Nakafero G; Avery AJ; Mamas MA; Abhishek A Association Between Gout Flare and Subsequent Cardiovascular Events Among Patients with Gout. JAMA 2022, 328, 440–450. 10.1001/jama.2022.11390. 35916846 PMC9346550

[38] Tedeschi SK; Huang W; Yoshida K; Solomon DH Risk of cardiovascular events in patients having had acute calcium pyrophosphate crystal arthritis. Ann. Rheum. Dis. 2022, 81, 1323–1329. 10.1136/annrheumdis-2022-222387. 35613842 PMC10043830

[39] Robinson PC; Terkeltaub R; Pillinger MH; Shah B; Karalis V; Karatza E; Liew D; Imazio M; Cornel JH; Thompson PL; Consensus Statement Regarding the Efficacy and Safety of Long-Term Low-Dose Colchicine in Gout and Cardiovascular Disease. Am. J. Med. 2022, 135, 32–38. 10.1016/j.amjmed.2021.07.025. 34416165 PMC8688259

[40] Terkeltaub RA; Sklar LA; Mueller H Neutrophil activation by inflammatory microcrystals of monosodium urate monohydrate utilizes pertussis toxin- insensitive and -sensitive pathways. J. Immunol. 1990, 144, 2719–2724. 2108211

[41] Onello E; Traynor-Kaplan A; Sklar L; Terkeltaub R Mechanism of neutrophil activation by an unopsonized inflammatory particulate. Monosodium urate crystals induce pertussis toxin-insensitive hydrolysis of phosphatidylinositol 4,5-bisphosphate. J. Immunol. 1991, 146, 4289–4294. 1645760

[42] Terkeltaub R; Solan J; Barry MJr Santoro D; Bokoch GM Role of the mevalonate pathway of isoprenoid synthesis in IL-8 generation by activated monocytic cells. J. Leukoc. Biol. 1994, 55, 749–755. 10.1002/jlb.55.6.749. 8195701

[43] Liu R; Aupperle K; Terkeltaub R Src family protein tyrosine kinase signaling mediates monosodiumurate crystal-induced IL-8 expression by monocytic THP-1 cells. J. Leukoc. Biol. 2001, 70, 961–968. 11739559

[44] Liu R; Lioté F; Rose DM; Merz D; Terkeltaub R Proline-rich tyrosine kinase 2 and Src kinase signaling transduce monosodium urate crystal-induced nitric oxide production and matrix metalloproteinase 3 expression in chondrocytes. Arthritis Rheum. 2004, 50, 247–258. 10.1002/art.11486. 14730623

[45] Liu R; O’Connell M; Johnson K; Pritzker K; Mackman N; Terkeltaub R Extracellular signal-regulated kinase 1/extracellular signal-regulated kinase 2 mitogen-activated protein kinase signaling and activation of activator protein 1 and nuclear factor kappaB transcription factors play central roles in interleukin-8 expression stimulated by monosodium urate monohydrate and calcium pyrophosphate crystals in monocytic cells. Arthritis Rheum. 2000, 43, 1145–1155. 10.1002/1529-0131(200005)43:5<1145::AID-ANR25>3.0.CO;2-T. 10817569

[46] Cobo I; Cheng A; Murillo-Saich J; Coras R; Torres A; Abe Y; Lana AJ; Schlachetzki J; Ru L-B; Terkeltaub R; Monosodium urate crystals regulate a unique JNK-dependent macrophage metabolic and inflammatory response. Cell Rep. 2022, 38, 110489. 10.1016/j.celrep.2022.110489. 35263587 PMC8989403

[47] Cobo I; Murillo-Saich J; Alishala M; Calderon S; Coras R; Hemming B; Inkum F; Rosas F; Takei R; Spann N; Particle uptake by macrophages triggers bifurcated transcriptional pathways that differentially regulate inflammation and lysosomal gene expression. Immunity 2025, 58, 826–842.e8. 10.1016/j.immuni.2025.02.023. 40118070 PMC12093573

[48] Wang Y; Viollet B; Terkeltaub R; Ru L-B AMP-activated protein kinase suppresses urate crystal-induced inflammation and transduces colchicine effects in macrophages. Ann. Rheum. Dis. 2016, 75, 286–294. 10.1136/annrheumdis-2014-206074. 25362043 PMC4417082

[49] McWherter C; Choi YJ; Serrano RL; Mahata SK; Terkeltaub R; Ru L-B Arhalofenate acid inhibits monosodium urate crystal-induced inflammatory responses through activation of AMP-activated protein kinase (AMPK) signaling. Arthritis Res. Ther. 2018, 20, 204. 10.1186/s13075-018-1699-4. 30189890 PMC6127987

[50] Vazirpanah N; Ottria A; van der Linden M; Wichers CGK; Schuiveling M; van Lochem E; Phipps-Green A; Merriman T; Zimmermann M; Jansen M; mTOR inhibition by metformin impacts monosodium urate crystal-induced inflammation and cell death in gout: A prelude to a new add-on therapy? Ann. Rheum. Dis. 2019, 78, 663–671. 10.1136/annrheumdis-2018-214656. 30814053

[51] Liu Z; Chu A; Bai Z; Yang C Nobiletin ameliorates monosodium urate-induced gouty arthritis in mice by enhancing AMPK/mTOR-mediated autophagy to inhibit NF-κB/NLRP3 inflammasome activation. Immunol. Lett. 2025, 274, 106982. 10.1016/j.imlet.2025.106982. 39965668

[52] Wang S; Li H; Yuan M; Fan H; Cai Z. Role of AMPK in autophagy. Front. Physiol. 2022, 13, 1015500. 10.3389/fphys.2022.1015500. 36505072 PMC9732440

[53] Park JM; Lee DH; Kim DH Redefining the role of AMPK in autophagy and the energy stress response. Nat. Commun. 2023, 14, 2994. 10.1038/s41467-023-38401-z. 37225695 PMC10209092

[54] Li J; Zhang B; Liu WX; Lu K; Pan H; Wang T; Oh CD; Yi D; Huang J; Zhao L; Metformin limits osteoarthritis development and progression through activation of AMPK signalling. Ann. Rheum. Dis. 2020, 79, 635–645. 10.1136/annrheumdis-2019-216713. 32156705 PMC7213329

[55] Yu W; Ru L-B; Stevens S; Damanahalli JK; Terkeltaub R RAGE signaling mediates post-injury arterial neointima formation by suppression of liver kinase B1 and AMPK activity. Atherosclerosis 2012, 222, 417–425. 10.1016/j.atherosclerosis.2012.04.001. 22552116 PMC3361645

[56] Marrugo J; Santacroce LM; Paudel ML; Fukui S; Turchin A; Tedeschi SK; Solomon DH Gout risk in adults with pre-diabetes initiating metformin. Ann. Rheum. Dis. 2024, 83, 1368–1374. 10.1136/ard-2024-225652. 38749572 PMC11442121

[57] Veenstra F; Verhoef LM; Opdam M; den Broeder AA; Kwok WY; Meek IL; van den Ende CHM; Flendrie M; van Herwaarden N Effect of metformin use on clinical outcomes and serum urate in gout patients with diabetes mellitus: A retrospective cohort study. BMC Rheumatol. 2022, 6, 27. 10.1186/s41927-022-00261-3. 35637534 PMC9153141

[58] Yokose C; McCormick N; Abhishek A; Dalbeth N; Pascart T; Lioté F; Gaffo A; FitzGerald J; Terkeltaub R; Sise ME; The clinical benefits of sodium-glucose cotransporter type 2 inhibitors in people with gout. Nat. Rev. Rheumatol. 2024, 20, 216–231. 10.1038/s41584-024-01092-x. 38472344 PMC13186535

[59] McCormick N; Yokose C; Lu N; Wexler DJ; Aviña-Zubieta JA; De Vera MA; McCoy RG; Choi HK Sodium-Glucose Cotransporter-2 Inhibitors vs Sulfonylureas for Gout Prevention Among Patients with Type 2 Diabetes Receiving Metformin. JAMA Intern. Med. 2024, 184, 650–660. 10.1001/jamainternmed.2024.0376. 38619822 PMC11019449

[60] Bousoik E; Qadri M; Elsaid KA CD44 Receptor Mediates Urate Crystal Phagocytosis by Macrophages and Regulates Inflammation in A Murine Peritoneal Model of Acute Gout. Sci. Rep. 2020, 10, 5748. 10.1038/s41598-020-62727-z. 32238827 PMC7113258

[61] Tang H; Xiao Y; Qian L; Wang Z; Lu M; Yao N; Zhou T; Tian F; Cao L; Zheng P; Mechanistic insights into the C-type lectin receptor CLEC12A-mediated immune recognition of monosodium urate crystal. J. Biol. Chem. 2024, 300, 105765. 10.1016/j.jbc.2024.105765. 38367667 PMC10959670

[62] Ru L-B; Scott P; Sydlaske A; Rose DM; Terkeltaub R Innate immunity conferred by Toll-like receptors 2 and 4 and myeloid differentiation factor 88 expression is pivotal to monosodium urate monohydrate crystal-induced inflammation. Arthritis Rheum. 2005, 52, 2936–2946. 10.1002/art.21238. 16142712

[63] Scott P; Ma H; Viriyakosol S; Terkeltaub R; Ru L-B Engagement of CD14 mediates the inflammatory potential of monosodium urate crystals. J. Immunol. 2006, 177, 6370–6378. 10.4049/jimmunol.177.9.6370. 17056568

[64] Alaswad A; Cabău G; Crişan TO; Zhou L; Zoodsma M; Botey-Bataller J; Li W; Pamfil C; Netea MG; Merriman T; Integrative analysis reveals the multilateral inflammatory Mechanisms of CD14 monocytes in gout. Ann. Rheum. Dis. 2025, 84, 1253–1263. 10.1016/j.ard.2025.01.046. 40023733

[65] Wang Z; Zhao Y; Phipps-Green A; Ru L-B; Ceponis A; Boyle DL; Wang J; Merriman TR; Wang W; Terkeltaub R Differential DNA Methylation of Networked Signaling, Transcriptional, Innate and Adaptive Immunity, and Osteoclastogenesis Genes and Pathways in Gout. Arthritis Rheumatol. 2020, 72, 802–814. 10.1002/art.41173. 31738005 PMC7323903

[66] Straton AR; Kischkel B; Crișan TO; Joosten LAB. Epigenomic Reprogramming in Gout. Gout Urate Cryst. Depos. Dis. 2024, 2, 325–338. 10.3390/gucdd2040023.

[67] Major TJ; Takei R; Matsuo H; Leask MP; Sumpter NA; Topless RK; Shirai Y; Wang W; Cadzow MJ; Phipps-Green AJ; A genome-wide association analysis reveals new pathogenic pathways in gout. Nat. Genet. 2024, 56, 2392–2406. 10.1038/s41588-024-01921-5. 39406924

[68] Agrawal M; Niroula A; Cunin P; McConkey M; Shkolnik V; Kim PG; Wong WJ; Weeks LD; Lin AE; Miller PG; TET2-mutant clonal hematopoiesis and risk of gout. Blood 2022, 140, 1094–1103. 10.1182/blood.2022015384. 35714308 PMC9461470

[69] Merriman TR; Joosten LAB CHIP and gout: Trained immunity? Blood 2022, 140, 1054–1056. 10.1182/blood.2022017212. 36074537

[70] Cobo I; Murillo-Saich J; Alishala M; Guma M Epigenetic and Metabolic Regulation of Macrophages during Gout. Gout Urate Cryst. Depos. Dis. 2023, 1, 137–151. 10.3390/gucdd1030013. PMC13492561 42626427

[71] Gu H; Yu H; Qin L; Yu H; Song Y; Chen G; Zhao D; Wang S; Xue W; Wang L; MSU crystal deposition contributes to inflammation and immune responses in gout remission. Cell Rep. 2023, 42, 113139. 10.1016/j.celrep.2023.113139. 37756161

[72] Dalbeth N; Pool B; Gamble GD; Smith T; Callon KE; McQueen FM; Cornish J Cellular characterization of the gouty tophus: A quantitative analysis. Arthritis Rheum. 2010, 62, 1549–1556. 10.1002/art.27356. 20131281

[73] Dalbeth N; Smith T; Nicolson B; Clark B; Callon K; Naot D; Haskard DO; McQueen FM; Reid IR; Cornish J Enhanced osteoclastogenesis in patients with tophaceous gout: Urate crystals promote osteoclast development through interactions with stromal cells. Arthritis Rheum. 2008, 58, 1854–1865. 10.1002/art.23488. 18512794

[74] Elsaid K; Merriman TR; Rossitto LA; Ru L-B; Karsh J; Phipps-Green A; Jay GD; Elsayed S; Qadri M; Miner M; Amplification of Inflammation by Lubricin Deficiency Implicated in Incident, Erosive Gout Independent of Hyperuricemia. Arthritis Rheumatol. 2023, 75, 794–805. 10.1002/art.42413. 36457235 PMC10191887

[75] Elsaid KA; Jay GD; Ru L-B; Terkeltaub R Proteoglycan 4 (PRG4)/Lubricin and the Extracellular Matrix in Gout. Gout Urate Cryst. Depos. Dis. 2023, 1, 122–136. 10.3390/gucdd1030012.

[76] Abhishek A; Neogi T; Choi H; Doherty M; Rosenthal AK; Terkeltaub R Review: Unmet Needs and the Path Forward in Joint Disease Associated with Calcium Pyrophosphate Crystal Deposition. Arthritis Rheumatol. 2018, 70, 1182–1191. 10.1002/art.40517. 29609209 PMC6105380

[77] Pascart T; Filippou G; Lioté F; Sirotti S; Jauffret C; Abhishek A Calcium pyrophosphate deposition disease. Lancet Rheumatol. 2024, 6, e791–e804. 10.1016/S2665-9913(24)00122-X. 39089298

[78] Terkeltaub R; Rosenbach M; Fong F; Goding J Causal link between nucleotide pyrophosphohydrolase overactivity and increased intracellular inorganic pyrophosphate generation demonstrated by transfection of cultured fibroblasts and osteoblasts with plasma cell membrane glycoprotein-1. Relevance to calcium pyrophosphate dihydrate deposition disease. Arthritis Rheum. 1994, 37, 934–941. 10.1002/art.1780370624. 8003067

[79] Huang R; Rosenbach M; Vaughn R; Provvedini D; Rebbe N; Hickman S; Goding J; Terkeltaub R Expression of the murine plasma cell nucleotide pyrophosphohydrolase PC-1 is shared by human liver, bone, and cartilage cells. Regulation of PC-1 expression in osteosarcoma cells by transforming growth factor-beta. J. Clin. Investig. 1994, 94, 560–567. 10.1172/JCI117370. 8040311 PMC296131

[80] Johnson K; Moffa A; Chen Y; Pritzker K; Goding J; Terkeltaub R Matrix vesicle plasma cell membrane glycoprotein-1 regulates mineralization by murine osteoblastic MC3T3 cells. J. Bone Miner. Res. 1999, 14, 883–892. 10.1359/jbmr.1999.14.6.883. 10352096

[81] Johnson K; Hashimoto S; Lotz M; Pritzker K; Goding J; Terkeltaub R Up-regulated expression of the phosphodiesterase nucleotide pyrophosphatase family member PC-1 is a marker and pathogenic factor for knee meniscal cartilage matrix calcification. Arthritis Rheum. 2001, 44, 1071–1081. 10.1002/1529-0131(200105)44:5<1071::AID-ANR187>3.0.CO;2-3. 11352238

[82] Anderson HC; Harmey D; Camacho NP; Garimella R; Sipe JB; Tague S; Bi X; Johnson K; Terkeltaub R; Millán JL Sustained osteomalacia of long bones despite major improvement in other hypophosphatasia-related mineral deficits in tissue nonspecific alkaline phosphatase/nucleotide pyrophosphatase phosphodiesterase 1 double-deficient mice. Am. J. Pathol. 2005, 166, 1711–1720. 10.1016/S0002-9440(10)62481-9. 15920156 PMC1602415

[83] Harmey D; Hessle L; Narisawa S; Johnson KA; Terkeltaub R; Millán JL Concerted regulation of inorganic pyrophosphate and osteopontin by akp2, enpp1, and ank: An integrated model of the pathogenesis of mineralization disorders. Am. J. Pathol. 2004, 164, 1199–1209. 10.1016/S0002-9440(10)63208-7. 15039209 PMC1615351

[84] Johnson K; Goding J; Van Etten D; Sali A; Hu SI; Farley D; Krug H; Hessle L; Millán JL; Terkeltaub R Linked deficiencies in extracellular PP(i) and osteopontin mediate pathologic calcification associated with defective PC-1 and ANK expression. J. Bone Miner. Res. 2003, 18, 994–1004. 10.1359/jbmr.2003.18.6.994. 12817751

[85] Hessle L; Johnson KA; Anderson HC; Narisawa S; Sali A; Goding JW; Terkeltaub R; Millan JL Tissue-nonspecific alkaline phosphatase and plasma cell membrane glycoprotein-1 are central antagonistic regulators of bone mineralization. Proc. Natl. Acad. Sci. USA 2002, 99, 9445–9449. 10.1073/pnas.142063399. 12082181 PMC123160

[86] Johnson KA; Hessle L; Vaingankar S; Wennberg C; Mauro S; Narisawa S; Goding JW; Sano K; Millan JL; Terkeltaub R Osteoblast tissue-nonspecific alkaline phosphatase antagonizes and regulates PC-1. Am. J. Physiol. Regul. Integr. Comp. Physiol. 2000, 279, R1365–R1377. 10.1152/ajpregu.2000.279.4.R1365. 11004006

[87] Johnson K; Terkeltaub R Upregulated ank expression in osteoarthritis can promote both chondrocyte MMP-13 expression and calcification via chondrocyte extracellular PPi excess. Osteoarthr. Cartil. 2004, 12, 321–335. 10.1016/j.joca.2003.12.004. 15023384

[88] Nitschke Y; Baujat G; Botschen U; Wittkampf T; du Moulin M; Stella J; Le Merrer M; Guest G; Lambot K; Tazarourte-Pinturier MF; Generalized arterial calcification of infancy and pseudoxanthoma elasticum can be caused by mutations in either ENPP1 or ABCC6. Am. J. Hum. Genet. 2012, 90, 25–39. 10.1016/j.ajhg.2011.11.020. 22209248 PMC3257960

[89] Rutsch F; Nitschke Y; Terkeltaub R Genetics in arterial calcification: Pieces of a puzzle and cogs in a wheel. Circ. Res. 2011, 109, 578–592. 10.1161/CIRCRESAHA.111.247965. 21852556 PMC3248761

[90] Szeri F; Niaziorimi F; Donnelly S; Fariha N; Tertyshnaia M; Patel D; Lundkvist S; van de Wetering K The Mineralization Regulator ANKH Mediates Cellular Efflux of ATP, Not Pyrophosphate. J. Bone Miner. Res. 2022, 37, 1024–1031. 10.1002/jbmr.4528. 35147247 PMC9098669

[91] Zhang Y; Johnson K; Russell RG; Wordsworth BP; Carr AJ; Terkeltaub RA; Brown MA Association of sporadic chondrocalcinosis with a −4-basepair G-to-A transition in the 5'-untranslated region of ANKH that promotes enhanced expression of ANKH protein and excess generation of extracellular inorganic pyrophosphate. Arthritis Rheum. 2005, 52, 1110–1117. 10.1002/art.20978. 15818664

[92] Abhishek A; Doherty S; Maciewicz R; Muir K; Zhang W; Doherty M; Valdes AM The association between ANKH promoter polymorphism and chondrocalcinosis is independent of age and osteoarthritis: Results of a case-control study. Arthritis Res Ther. 2014, 16, R25. 10.1186/ar4453. 24467728 PMC3978851

[93] Jacob J, Aggarwal A, Aggarwal A, Bhattacharyya S, Kumar V, Sharma V, Sahni D Senescent chondrogenic progenitor cells derived from articular cartilage of knee osteoarthritis patients contributes to senescence-associated secretory phenotype via release of IL-6 and IL-8. Acta histochemica, 2022. 124, 151867. 10.1016/j.acthis.2022.151867 35192993

[94] Jeon OH, Kim C, Laberge RM, Demaria M, Rathod S, Vasserot AP, Chung JW, Kim DH, Poon Y, David N, Baker DJ, van Deursen JM, Campisi J, Elisseeff JH Local clearance of senescent cells attenuates the development of post-traumatic osteoarthritis and creates a pro-regenerative environment. Nature medicine, 2017. 23, 775–781. 10.1038/nm.4324 PMC5785239 28436958

[95] Szeri F, Lundkvist S, Donnelly S, Engelke UFH, Rhee K, Williams CJ, Sundberg JP, Wevers RA, Tomlinson RE, Jansen RS, & van de Wetering K The membrane protein ANKH is crucial for bone mechanical performance by mediating cellular export of citrate and ATP. PLoS genetics, 16, e1008884. 10.1371/journal.pgen.1008884 PMC7371198 32639996

[96] James EN, Teh MT, Li Y, Wagner-Bock C, Al-Khateeb ZF, Karen-Ng LP, Roberts T, Synchyshyn L, Lewis A, OĽoghlen A, Silver A, Michael-Titus AT, Bennett M, Bundy JG, Mycielska ME, & Parkinson EK Membrane transporter progressive ankylosis protein homologue (*ANKH/Ank*) partially mediates senescence-derived extracellular citrate and is regulated by DNA damage, inflammation, and ageing. Frontiers in aging, 2025. 6, 1583288. 10.3389/fragi.2025.1583288 40535023 PMC12174118

[97] Richter E, Lohmann CH, Dell'Accio F, Goettsch C, Bertrand J. Sortilin Is Upregulated in Osteoarthritis-Dependent Cartilage Calcification and Associated with Cellular Senescence. Int J Mol Sci. 2023;24:12343. doi:10.3390/ijms241512343. 37569721 PMC10418692

[98] Doherty M, Hamilton E, Henderson J, Misra H, & Dixey J Familial chondrocalcinosis due to calcium pyrophosphate dihydrate crystal deposition in English families. 1991. British journal of rheumatology, 30, 10–15. 10.1093/rheumatology/30.1.10 1846765

[99] Olmez U; Ryan LM; Kurup IV; Rosenthal AK Insulin-like growth factor-1 suppresses pyrophosphate elaboration by transforming growth factor beta1-stimulated chondrocytes and cartilage. Osteoarthr. Cartil. 1994, 2, 149–154. 10.1016/s1063-4584(05)80065-2. 11550674

[100] Terkeltaub RA; Johnson K; Rohnow D; Goomer R; Burton D; Deftos LJ Bone morphogenetic proteins and bFGF exert opposing regulatory effects on PTHrP expression and inorganic pyrophosphate elaboration in immortalized murine endochondral hypertrophic chondrocytes (MCT cells). J. Bone Miner. Res. 1998, 13, 931–941. 10.1359/jbmr.1998.13.6.931. 9626624

[101] Terkeltaub R; Lotz M; Johnson K; Deng D; Hashimoto S; Goldring MB; Burton D; Deftos LJ Parathyroid hormone-related proteins is abundant in osteoarthritic cartilage, and the parathyroid hormone-related protein 1–173 isoform is selectively induced by transforming growth factor beta in articular chondrocytes and suppresses generation of extracellular inorganic pyrophosphate. Arthritis Rheum. 1998, 41, 2152–2164. 10.1002/1529-0131(199812)41:12<2152::AID-ART10>3.0.CO;2-X. 9870872

[102] Lotz M; Rosen F; McCabe G; Quach J; Blanco F; Dudler J; Solan J; Goding J; Seegmiller JE; Terkeltaub R Interleukin 1 beta suppresses transforming growth factor-induced inorganic pyrophosphate (PPi) production and expression of the PPi-generating enzyme PC-1 in human chondrocytes. Proc. Natl. Acad. Sci. USA 1995, 92, 10364–10368. 10.1073/pnas.92.22.10364. 7479785 PMC40797

[103] Rosen F; McCabe G; Quach J; Solan J; Terkeltaub R; Seegmiller JE; Lotz M Differential effects of aging on human chondrocyte responses to transforming growth factor beta: Increased pyrophosphate production and decreased cell proliferation. Arthritis Rheum. 1997, 40, 1275–1281. doi: 10.1002/1529-0131(199707)40:7<1275::AID-ART12>3.0.CO;2-H. 9214428

[104] Hashimoto S; Ochs RL; Rosen F; Quach J; McCabe G; Solan J; Seegmiller JE; Terkeltaub R; Lotz M Chondrocyte-derived apoptotic bodies and calcification of articular cartilage. Proc. Natl. Acad. Sci. USA 1998, 95, 3094–3099. 10.1073/pnas.95.6.3094. 9501221 PMC19700

[105] Johnson K; Vaingankar S; Chen Y; Moffa A; Goldring MB; Sano K; Jin-Hua P; Sali A; Goding J; Terkeltaub R Differential mechanisms of inorganic pyrophosphate production by plasma cell membrane glycoprotein-1 and B10 in chondrocytes. Arthritis Rheum. 1999, 42, 1986–1997. 10.1002/1529-0131(199909)42:9<1986::AID-ANR26>3.0.CO;2-O. 10513816

[106] Johnson K; Farley D; Hu SI; Terkeltaub R One of two chondrocyte-expressed isoforms of cartilage intermediate-layer protein functions as an insulin-like growth factor 1 antagonist. Arthritis Rheum. 2003, 48, 1302–1314. 10.1002/art.10927. 12746903

[107] Takei R; Rosenthal A; Pascart T; Reynolds RJ; Neogi T; Terkeltaub R; Tedeschi SK; Merriman TR Genome-wide association study in chondrocalcinosis reveals ENPP1 as a candidate therapeutic target in calcium pyrophosphate deposition disease. Ann. Rheum. Dis. 2025, 84, 1023–1032. 10.1016/j.ard.2025.04.002. 40483170 PMC12248263

[108] Nassir M; Mirza S; Arad U; Lee S; Rafehi M; Yaw Attah I; Renn C; Zimmermann H; Pelletier J; Sévigny J; Adenine-(methoxy)-ethoxy-P_α,α_-dithio-triphosphate inhibits pathologic calcium pyrophosphate deposition in osteoarthritic human chondrocytes. Org. Biomol. Chem. 2019, 17, 9913–9923. 10.1039/c9ob02199j 31720670

[109] Ralph D; van de Wetering K; Uitto J; Li Q Inorganic Pyrophosphate Deficiency Syndromes and Potential Treatments for Pathologic Tissue Calcification. Am. J. Pathol. 2022, 192, 762–770. 10.1016/j.ajpath.2022.01.012. 35182493 PMC9088198

[110] Rutsch F; Vaingankar S; Johnson K; Goldfine I; Maddux B; Schauerte P; Kalhoff H; Sano K; Boisvert WA; Superti-Furga A; PC-1 nucleoside triphosphate pyrophosphohydrolase deficiency in idiopathic infantile arterial calcification. Am. J. Pathol. 2001, 158, 543–554. 10.1016/S0002-9440(10)63996-X. 11159191 PMC1850320

[111] Rutsch F; Ruf N; Vaingankar S; Toliat MR; Suk A; Höhne W; Schauer G; Lehmann M; Roscioli T; Schnabel D; Mutations in ENPP1 are associated with 'idiopathic' infantile arterial calcification. Nat. Genet. 2003, 34, 379–381. 10.1038/ng1221. 12881724

[112] Ruf N; Uhlenberg B; Terkeltaub R; Nürnberg P; Rutsch F The mutational spectrum of ENPP1 as arising after the analysis of 23 unrelated patients with generalized arterial calcification of infancy (GACI). Hum. Mutat. 2005, 25, 98. 10.1002/humu.9297. 15605415

[113] Rutsch F; Böyer P; Nitschke Y; Ruf N; Lorenz-Depierieux B; Wittkampf T; Weissen-Plenz G; Fischer RJ; Mughal Z; Gregory JW; Hypophosphatemia, hyperphosphaturia, and bisphosphonate treatment are associated with survival beyond infancy in generalized arterial calcification of infancy. Circ. Cardiovasc. Genet. 2008, 1, 133–140. 10.1161/CIRCGENETICS.108.797704. 20016754 PMC2794045

[114] Johnson K; Polewski M; van Etten D; Terkeltaub R Chondrogenesis mediated by PPi depletion promotes spontaneous aortic calcification in NPP1−/− mice. Arterioscler. Thromb. Vasc. Biol. 2005, 25, 686–691. 10.1161/01.ATV.0000154774.71187.f0. 15625282

[115] Johnson KA; Yao W; Lane NE; Naquet P; Terkeltaub RA Vanin-1 pantetheinase drives increased chondrogenic potential of mesenchymal precursors in ank/ank mice. Am. J. Pathol. 2008, 172, 440–453. 10.2353/ajpath.2008.070753. 18187567 PMC2312367

[116] Dammanahalli KJ; Stevens S; Terkeltaub R Vanin-1 pantetheinase drives smooth muscle cell activation in post-arterial injury neointimal hyperplasia. PLoS ONE 2012, 7, e39106. 10.1371/journal.pone.0039106. 22720042 PMC3374784

[117] Serrano RL; Yu W; Terkeltaub R Mono-allelic and bi-allelic ENPP1 deficiency promote post-injury neointimal hyperplasia associated with increased C/EBP homologous protein expression. Atherosclerosis 2014, 233, 493–502. 10.1016/j.atherosclerosis.2014.01.003. 24530784 PMC4386864

[118] Nitschke Y; Yan Y; Buers I; Kintziger K; Askew K; Rutsch F ENPP1-Fc prevents neointima formation in generalized arterial calcification of infancy through the generation of AMP. Exp. Mol. Med. 2018, 50, 1–12. 10.1038/s12276-018-0163-5. PMC6204430 30369595

[119] Tchernychev B; Nitschke Y; Chu D; Sullivan C; Flaman L; O’Brien K; Howe J; Cheng Z; Thompson D; Ortiz D; Inhibition of Vascular Smooth Muscle Cell Proliferation by ENPP1: The Role of CD73 and the Adenosine Signaling Axis. Cells 2024, 13, 1128. 10.3390/cells13131128. 38994980 PMC11240470

[120] Fuerst M; Bertrand J; Lammers L; Dreier R; Echtermeyer F; Nitschke Y; Rutsch F; Schäfer FK; Niggemeyer O; Steinhagen J; Calcification of articular cartilage in human osteoarthritis. Arthritis Rheum. 2009, 60, 2694–2703. 10.1002/art.24774. 19714647

[121] Jaabar IL; Foley B; Mezzetti A; Pillier F; Berenbaum F; Landoulsi J; Houard X Unraveling the Mechanisms of Hypertrophy-Induced Matrix Mineralization and Modifications in Articular Chondrocytes. Calcif. Tissue Int. 2024, 115, 269–282. 10.1007/s00223-024-01229-w. 38918254

[122] Jin Y; Cong Q; Gvozdenovic-Jeremic J; Hu J; Zhang Y; Terkeltaub R; Yang Y Enpp1 inhibits ectopic joint calcification and maintains articular chondrocytes by repressing hedgehog signaling. Development 2018, 145, dev164830. 10.1242/dev.164830. 30111653 PMC6176935

[123] Cronstein BN; Angle SR Purines and Adenosine Receptors in Osteoarthritis. Biomolecules 2023, 13, 1760. 10.3390/biom13121760. 38136631 PMC10741532

[124] Friedman B; Larranaga-Vera A; Castro CM; Corciulo C; Rabbani P; Cronstein BN Adenosine A2A receptor activation reduces chondrocyte senescence. FASEB J. 2023, 37, e22838. 10.1096/fj.202201212RR. 36884388 PMC11977601

[125] Friedman B; Corciulo C; Castro CM; Cronstein BN Adenosine A2A receptor signaling promotes FoxO associated autophagy in chondrocytes. Sci. Rep. 2021, 11, 968. 10.1038/s41598-020-80244-x. 33441836 PMC7806643

[126] Corciulo C; Castro CM; Coughlin T; Jacob S; Li Z; Fenyö D; Rifkin DB; Kennedy OD; Cronstein BN Intraarticular injection of liposomal adenosine reduces cartilage damage in established murine and rat models of osteoarthritis. Sci. Rep. 2020, 10, 13477. 10.1038/s41598-020-68302-w. 32778777 PMC7418027

[127] Castro CM; Corciulo C; Solesio ME; Liang F; Pavlov EV; Cronstein BN Adenosine A2A receptor (A2AR) stimulation enhances mitochondrial metabolism and mitigates reactive oxygen species-mediated mitochondrial injury. FASEB J. 2020, 34, 5027–5045. 10.1096/fj.201902459R. 32052890 PMC7202471

[128] Serrano RL; Chen LY; Lotz MK; Ru L-B; Terkeltaub R Impaired Proteasomal Function in Human Osteoarthritic Chondrocytes Can Contribute to Decreased Levels of SOX9 and Aggrecan. Arthritis Rheumatol. 2018, 70, 1030–1041. 10.1002/art.40456. 29457374 PMC6019618

[129] Husa M; Petursson F; Lotz M; Terkeltaub R; Ru L-B C/EBP homologous protein drives pro-catabolic responses in chondrocytes. Arthritis Res. Ther. 2013, 15, R218. 10.1186/ar4415. 24351550 PMC3978428

[130] Zhao X; Petursson F; Viollet B; Lotz M; Terkeltaub R; Ru L-B Peroxisome proliferator-activated receptor γ coactivator 1α and FoxO3A mediate chondroprotection by AMP-activated protein kinase. Arthritis Rheumatol. 2014, 66, 3073–3082. 10.1002/art.38791. 25047750 PMC4343036

[131] Petursson F; Husa M; June R; Lotz M; Terkeltaub R; Ru L-B Linked decreases in liver kinase B1 and AMP-activated protein kinase activity modulate matrix catabolic responses to biomechanical injury in chondrocytes. Arthritis Res. Ther. 2013, 15, R77. 10.1186/ar4254. 23883619 PMC3979085

[132] Terkeltaub R; Yang B; Lotz M; Ru L-B Chondrocyte AMP-activated protein kinase activity suppresses matrix degradation responses to proinflammatory cytokines interleukin-1β and tumor necrosis factor α. Arthritis Rheum. 2011, 63, 1928–1937. 10.1002/art.30333. 21400477 PMC3128233

[133] Johnson K; Svensson CI; Etten DV; Ghosh SS; Murphy AN; Powell HC; Terkeltaub R Mediation of spontaneous knee osteoarthritis by progressive chondrocyte ATP depletion in Hartley guinea pigs. Arthritis Rheum. 2004, 50, 1216–1225. 10.1002/art.20149. 15077304

[134] Johnson K; Jung A; Murphy A; Andreyev A; Dykens J; Terkeltaub R Mitochondrial oxidative phosphorylation is a downstream regulator of nitric oxide effects on chondrocyte matrix synthesis and mineralization. Arthritis Rheum. 2000, 43, 1560–1570. 10.1002/1529-0131(200007)43:7<1560::AID-ANR21>3.0.CO;2-S. 10902761

[135] Wang Y; Zhao X; Lotz M; Terkeltaub R; Ru L-B Mitochondrial biogenesis is impaired in osteoarthritis chondrocytes but reversible via peroxisome proliferator-activated receptor γ coactivator 1α. Arthritis Rheumatol. 2015, 67, 2141–2153. 10.1002/art.39182. 25940958 PMC4519411

[136] Chen LY; Wang Y; Terkeltaub R; Ru L-B Activation of AMPK-SIRT3 signaling is chondroprotective by preserving mitochondrial DNA integrity and function. Osteoarthr. Cartil. 2018, 26, 1539–1550. 10.1016/j.joca.2018.07.004. PMC6202232 30031925

[137] Li H; Ding X; Terkeltaub R; Lin H; Zhang Y; Zhou B; He K; Li K; Liu Z; Wei J; Exploration of metformin as novel therapy for osteoarthritis: Preventing cartilage degeneration and reducing pain behavior. Arthritis Res. Ther. 2020, 22, 34. 10.1186/s13075-020-2129-y. 32087740 PMC7036179

[138] van Galen I, Caron MMJ, van den Akker GGH, Welting TJM Drug repurposing for osteoarthritis disease modification in the Early 21st Century. Connect Tissue Res. 2025 Aug 2:1–9. doi: 10.1080/03008207.2025.2538562. 40751563

[139] Zhu Z; Huang JY; Ruan G; Cao P; Chen S; Zhang Y; Han W; Chen T; Cai X; Liu J; Metformin use and associated risk of total joint replacement in patients with type 2 diabetes: A population-based matched cohort study. CMAJ 2022, 194, E1672–E1684. 10.1503/cmaj.220952. 36535678 PMC9829054

[140] Lu CH; Chung CH; Lee CH; Hsieh CH; Hung YJ; Lin FH; Tsao CH; Hsieh PS; Chien WC Combination COX-2 inhibitor and metformin attenuate rate of joint replacement in osteoarthritis with diabetes: A nationwide, retrospective, matched-cohort study in Taiwan. PLoS ONE 2018, 13, e0191242. 10.1371/journal.pone.0191242. 29385156 PMC5791980

[141] Baker MC; Sheth K; Liu Y; Lu D; Lu R; Robinson WH Development of Osteoarthritis in Adults with Type 2 Diabetes Treated with Metformin vs a Sulfonylurea. JAMA Netw. Open. 2023, 6, e233646. 10.1001/jamanetworkopen.2023.3646. 36939700 PMC10028483

[142] Lai FTT; Yip BHK; Hunter DJ; Rabago DP; Mallen CD; Yeoh EK; Wong SYS; Sit RW Metformin use and the risk of total knee replacement among diabetic patients: A propensity-score-matched retrospective cohort study. Sci. Rep. 2022, 12, 11571. 10.1038/s41598-022-15871-7. 35798867 PMC9262887

[143] Wang Y; Hussain SM; Wluka AE; Lim YZ; Abram F; Pelletier JP; Martel-Pelletier J; Cicuttini FM Association between metformin use and disease progression in obese people with knee osteoarthritis: Data from the Osteoarthritis Initiative-a prospective cohort study. Arthritis Res. Ther. 2019, 21, 127. 10.1186/s13075-019-1915-x. 31126352 PMC6534888

[144] Neogi T; Nevitt M; Niu J; LaValley MP; Hunter DJ; Terkeltaub R; Carbone L; Chen H; Harris T; Kwoh K; Lack of association between chondrocalcinosis and increased risk of cartilage loss in knees with osteoarthritis: Results of two prospective longitudinal magnetic resonance imaging studies. Arthritis Rheum. 2006, 54, 1822–1828. 10.1002/art.21903. 16729275

[145] Latourte A; Rat AC; Ngueyon Sime W; Ea HK; Bardin T; Mazières B; Roux C; Guillemin F; Richette P Chondrocalcinosis of the Knee and the Risk of Osteoarthritis Progression: Data from the Knee and Hip Osteoarthritis Long-term Assessment Cohort. Arthritis Rheumatol. 2020, 72, 726–732. 10.1002/art.41186. 31804010

[146] Villiger PM; Terkeltaub R; Lotz M Monocyte chemoattractant protein-1 (MCP-1) expression in human articular cartilage. Induction by peptide regulatory factors and differential effects of dexamethasone and retinoic acid. J. Clin. Investig. 1992, 90, 488–496. 10.1172/JCI115885. 1365641 PMC443125

[147] Lotz M; Terkeltaub R; Villiger PM Cartilage and joint inflammation. Regulation of IL-8 expression by human articular chondrocytes. J. Immunol. 1992, 148, 466–473. 1729366

[148] Merz D; Liu R; Johnson K; Terkeltaub R IL-8/CXCL8 and growth-related oncogene alpha/CXCL1 induce chondrocyte hypertrophic differentiation. J. Immunol. 2003, 171, 4406–4415. 10.4049/jimmunol.171.8.4406. 14530367

[149] Cecil DL; David MR; Terkeltaub R; Ru L-B Role of interleukin-8 in PiT-1 expression and CXCR1-mediated inorganic phosphate uptake in chondrocytes. Arthritis Rheum. 2005, 52, 144–154. 10.1002/art.20748. 15641067

[150] Cecil DL; Johnson K; Rediske J; Lotz M; Schmidt AM; Terkeltaub R Inflammation-induced chondrocyte hypertrophy is driven by receptor for advanced glycation end products. J. Immunol. 2005, 175, 8296–8302. 10.4049/jimmunol.175.12.8296. 16339570

[151] Ru L-B; Terkeltaub R Chondrocyte innate immune myeloid differentiation factor 88-dependent signaling drives procatabolic effects of the endogenous Toll-like receptor 2/Toll-like receptor 4 ligands low molecular weight hyaluronan and high mobility group box chromosomal protein 1 in mice. Arthritis Rheum. 2010, 62, 2004–2012. 10.1002/art.27475. 20506365 PMC2902571

[152] Cecil DL; Appleton CT; Polewski MD; Mort JS; Schmidt AM; Bendele A; Beier F; Terkeltaub R The pattern recognition receptor CD36 is a chondrocyte hypertrophy marker associated with suppression of catabolic responses and promotion of repair responses to inflammatory stimuli. J. Immunol. 2009, 182, 5024–5031. 10.4049/jimmunol.0803603. 19342682 PMC2698125

[153] Johnson KA; Rose DM; Terkeltaub RA Factor XIIIA mobilizes transglutaminase 2 to induce chondrocyte hypertrophic differentiation. J. Cell Sci. 2008, 121 Pt 13, 2256–2264. 10.1242/jcs.011262. 18544639 PMC2615675

[154] Johnson KA; van Etten D; Nanda N; Graham RM; Terkeltaub RA Distinct transglutaminase 2-independent and transglutaminase 2-dependent pathways mediate articular chondrocyte hypertrophy. J. Biol. Chem. 2003, 278, 18824–18832. 10.1074/jbc.M301055200. 12606540

[155] Johnson KA; Terkeltaub RA External GTP-bound transglutaminase 2 is a molecular switch for chondrocyte hypertrophic differentiation and calcification. J. Biol. Chem. 2005, 280, 15004–15012. 10.1074/jbc.M500962200. 15691824

[156] Cecil DL; Terkeltaub R Transamidation by transglutaminase 2 transforms S100A11 calgranulin into a procatabolic cytokine for chondrocytes. J. Immunol. 2008, 180, 8378–8385. 10.4049/jimmunol.180.12.8378. 18523305 PMC2577366

[157] Huebner JL; Johnson KA; Kraus VB; Terkeltaub RA Transglutaminase 2 is a marker of chondrocyte hypertrophy and osteoarthritis severity in the Hartley guinea pig model of knee OA. Osteoarthr. Cartil. 2009, 17, 1056–1064. 10.1016/j.joca.2009.02.015. PMC3249463 19328881

[158] Johnson K; Hashimoto S; Lotz M; Pritzker K; Terkeltaub R Interleukin-1 induces pro-mineralizing activity of cartilage tissue transglutaminase and factor XIIIa. Am. J. Pathol. 2001, 159, 149–163. 10.1016/S0002-9440(10)61682-3. 11438463 PMC1850418

[159] Fan Y; Bian X; Meng X; Li L; Fu L; Zhang Y; Wang L; Zhang Y; Gao D; Guo X; Unveiling inflammatory and prehypertrophic cell populations as key contributors to knee cartilage degeneration in osteoarthritis using multi-omics data integration. Ann. Rheum. Dis. 2024, 83, 926–944. 10.1136/ard-2023-224420. 38325908 PMC11187367

[160] Sherwood J; Bertrand J; Nalesso G; Poulet B; Pitsillides A; Brandolini L; Karystinou A; De Bari C; Luyten FP; Pitzalis C; A homeostatic function of CXCR2 signalling in articular cartilage. Ann. Rheum. Dis. 2015, 74, 2207–2215. 10.1136/annrheumdis-2014-205546. 25135253 PMC4680121

[161] Johnson KA; Polewski M; Terkeltaub RA Transglutaminase 2 is central to induction of the arterial calcification program by smooth muscle cells. Circ. Res. 2008, 102, 529–537. 10.1161/CIRCRESAHA.107.154260. 18202319 PMC2652242

[162] Tang X; Peng Y; Jiang Z; Yu B; Peng D; Xie X; Li F; Ge Y Efferocytosis and its role in rheumatic diseases. Arthritis Rheumatol. 2025 Jun 2. doi: 10.1002/art.43268. Online ahead of print. 40452369

[163] Rose DM; Sydlaske AD; Agha-Babakhani A; Johnson K; Terkeltaub R Transglutaminase 2 limits murine peritoneal acute gout-like inflammation by regulating macrophage clearance of apoptotic neutrophils. Arthritis Rheum. 2006, 54, 3363–3371. 10.1002/art.22137. 17009310

[164] Boisvert WA; Rose DM; Boullier A; Quehenberger O; Sydlaske A; Johnson KA; Curtiss LK; Terkeltaub R Leukocyte transglutaminase 2 expression limits atherosclerotic lesion size. Arterioscler Thromb. Vasc. Biol. 2006, 26, 563–569. 10.1161/01.ATV.0000203503.82693.c1. 16410462

[165] Serrano RL; Yu W; Graham RM; Bryan RL; Terkeltaub R A vascular smooth muscle cell X-box binding protein 1 and transglutaminase 2 regulatory circuit limits neointimal hyperplasia. PLoS ONE 2019, 14, e0212235. 10.1371/journal.pone.0212235. 30943188 PMC6447169

[166] Schauer C; Janko C; Munoz LE; Zhao Y; Kienhöfer D; Frey B; Lell M; Manger B; Rech J; Naschberger E; Aggregated neutrophil extracellular traps limit inflammation by degrading cytokines and chemokines. Nat. Med. 2014, 20, 511–517. 10.1038/nm.3547. 24784231

[167] Liu L; Shan L; Wang H; Schauer C; Schoen J; Zhu L; Lu C; Wang Z; Xue Y; Wu H; Neutrophil Extracellular Trap-Borne Elastase Prevents Inflammatory Relapse in Intercritical Gout. Arthritis Rheumatol. 2023, 75, 1039–1047. 10.1002/art.42431. 36575650

[168] Hahn J; Schauer C; Czegley C; Kling L; Petru L; Schmid B; Weidner D; Reinwald C; Biermann MHC; Blunder S; Aggregated neutrophil extracellular traps resolve inflammation by proteolysis of cytokines and chemokines and protection from antiproteases. FASEB J. 2019, 33, 1401–1414. 10.1096/fj.201800752R. 30130433 PMC6355082

